# Global Increase in Circular RNA Levels in Myotonic Dystrophy

**DOI:** 10.3389/fgene.2019.00649

**Published:** 2019-07-18

**Authors:** Karol Czubak, Katarzyna Taylor, Agnieszka Piasecka, Krzysztof Sobczak, Katarzyna Kozlowska, Anna Philips, Saam Sedehizadeh, J. David Brook, Marzena Wojciechowska, Piotr Kozlowski

**Affiliations:** ^1^Department of Molecular Genetics, Institute of Bioorganic Chemistry, Polish Academy of Sciences, Poznan, Poland; ^2^Laboratory of Gene Therapy, Department of Gene Expression, Institute of Molecular Biology and Biotechnology, Faculty of Biology, Adam Mickiewicz University, Poznan, Poland; ^3^European Center for Bioinformatics and Genomics, Institute of Bioorganic Chemistry, Polish Academy of Sciences, Poznan, Poland; ^4^Queen’s Medical Centre, School of Life Sciences, University of Nottingham, Nottingham, United Kingdom

**Keywords:** circular RNAs (circRNAs), myotonic dystrophy type 1 (DM1), circRNA biogenesis, CIRI2, droplet digital PCR (ddPCR), DM1 biomarkers

## Abstract

Splicing aberrations induced as a consequence of the sequestration of muscleblind-like splicing factors on the dystrophia myotonica protein kinase transcript, which contains expanded CUG repeats, present a major pathomechanism of myotonic dystrophy type 1 (DM1). As muscleblind-like factors may also be important factors involved in the biogenesis of circular RNAs (circRNAs), we hypothesized that the level of circRNAs would be decreased in DM1. To test this hypothesis, we selected 20 well-validated circRNAs and analyzed their levels in several experimental systems (e.g., cell lines, DM muscle tissues, and a mouse model of DM1) using droplet digital PCR assays. We also explored the global level of circRNAs using two RNA-Seq datasets of DM1 muscle samples. Contrary to our original hypothesis, our results consistently showed a global increase in circRNA levels in DM1, and we identified numerous circRNAs that were increased in DM1. We also identified many genes (including muscle-specific genes) giving rise to numerous (>10) circRNAs. Thus, this study is the first to show an increase in global circRNA levels in DM1. We also provided preliminary results showing the association of circRNA level with muscle weakness and alternative splicing changes that are biomarkers of DM1 severity.

## Introduction

Myotonic dystrophy type 1 (*dystrophia myotonica 1*, DM1, OMIM: 160900) is the most common form of adult-onset muscular dystrophy, affecting approximately 1 in 8,000 people worldwide. DM1 is an autosomal dominant disorder caused by an expansion of CTG repeats in the 3′ untranslated region of the *dystrophia myotonica protein kinase* (*DMPK*) gene (Brook et al., 1992; Fu et al., 1992; Mahadevan et al., 1992). Unaffected individuals have between 5 and ∼34 repeats, whereas in DM1 patients, the triplet repeat is expanded, often to hundreds or even thousands of copies (Brook et al., 1992). The pathogenesis of DM1 is strongly linked to the expression of mutation-containing transcripts and is manifested through the nuclear accumulation of mutant transcripts in characteristic foci (Taneja et al., 1995). The presence of these mutant transcripts causes the sequestration of muscleblind-like (MBNL) proteins [including MBNL1, the main MBNL family protein in muscles (Fardaei et al., 2002; Konieczny et al., 2014), MBNL2, and MBNL3], which normally regulate alternative splicing of pre-messenger RNAs (pre-mRNAs) encoding proteins critical for skeletal, cardiac, and nervous system function (Miller et al., 2000; Pascual et al., 2006). Thus, their sequestration and functional insufficiency result in aberrant alternative splicing of many target genes. For example, mis-splicing of the *CLCN1* exon 7, the *INSR* exon 11, and the *BIN1* exon 11 were shown to be associated with reduced chloride conductance, lower insulin responsiveness, and muscle weakness, respectively (Philips et al., 1998; Savkur et al., 2001; Mankodi et al., 2002; Ho et al., 2004; Fugier et al., 2011). A pathomechanism similar to that observed in DM1 was also proposed for myotonic dystrophy type 2 (*dystrophia myotonica 2*, DM2, OMIM: 602668), a disease caused by an expansion of CCTG repeats in the first intron of the *CCHC-type zinc finger nucleic acid binding protein* gene (Liquori et al., 2001). However, in this study, we mainly focused on DM1.

The results of a recent study suggest that in addition to a function in alternative splicing, MBNLs may play an important role in the biogenesis of a recently recognized class of RNA molecules called circular RNAs (circRNAs) (Ashwal-Fluss et al., 2014). Unlike other types of RNA, circRNAs are very stable molecules. Due to the low expression level of the initially identified circRNAs, they were considered byproducts of aberrant RNA splicing. However, with the dissemination of RNA-Seq technology, research has revealed that circRNAs are abundant among a variety of transcriptomes (Memczak et al., 2013; Salzman et al., 2013). Although the levels of most circRNAs are low, there are examples of circRNAs with levels comparable with or higher than those of their linear counterparts (Jeck et al., 2013). Most circRNAs are encoded by protein-coding genes and derived from their exons, which may indicate that transcription of circRNAs is directed by RNA polymerase II and that their biogenesis is mediated by the spliceosome. In the majority of cases, head-to-tail junctions of circular transcripts are flanked by canonical splice sites (Ashwal-Fluss et al., 2014; Starke et al., 2015). Reportedly, the formation of circRNAs may occur both post-transcriptionally and cotranscriptionally (Wilusz and Sharp, 2013; Ashwal-Fluss et al., 2014; Kramer et al., 2015), and their biogenesis competes with the formation of linear transcripts (mRNA). The mechanisms of this competition are tissue specific and conserved from flies to humans (Ashwal-Fluss et al., 2014; Rybak-Wolf et al., 2015). To date, no function has been assigned for the vast majority of circRNAs, with exceptions such as circCDR1as, *Sry* circRNA, or circHIPK3 (hsa_circ_0000284), which can act as microRNA sponges (Hansen et al., 2011; Memczak et al., 2013; Zheng et al., 2016). Other functions, such as involvement in protein and/or RNA transport (Memczak et al., 2013), regulating synaptic functions in neural tissue (You et al., 2015), or acting as templates for translation of functional peptides [e.g., (Li and Lytton, 1999)], have also been proposed for circRNAs.

The precise mechanism of circRNA generation remains unknown. However, several mechanisms of circRNA biogenesis have been proposed (Salzman et al., 2012; Jeck et al., 2013; Salzman et al., 2013). All of these proposed mechanisms assume the generation of circRNAs by head-to-tail splicing (back-splicing). One of the proposed mechanisms suggests that RNA-binding proteins (RBPs), which bind to specific motifs in introns flanking circRNA-coding exons, play an important role in circRNA biogenesis (Ashwal-Fluss et al., 2014; Conn et al., 2015). Back-splicing is facilitated by the interaction between RBPs, which bring the introns closer together. The *Drosophila* Mbl protein (ortholog of human MBNLs) may be a circRNA-biogenesis RBP (Ashwal-Fluss et al., 2014). Interestingly, one of Mbl-regulated circRNAs is circMBNL1/circMbl, a circRNA generated from the second exon of the *MBNL1/Mbl* gene. The introns flanking this circRNA contain highly conserved MBNL/Mbl-binding motifs. Furthermore, the exogenous expression of Mbl stimulates circRNA production from endogenous MBNL1/Mbl transcripts in both humans and flies. Mbl-binding sequences in both introns are necessary, suggesting that Mbl induces circularization by bridging the two flanking introns. Importantly, downregulation of Mbl in both fly cell culture and fly neural tissue leads to a significant decrease in circMbl level, whereas the elevated level of Mbl increases the level of circMbl as well as other circRNAs, suggesting a general role for MBNLs/Mbl in circRNA biogenesis (Ashwal-Fluss et al., 2014).

In this work, we aimed to test the level of circRNAs in DM1. Since MBNL proteins may be involved in circRNA biogenesis (Ashwal-Fluss et al., 2014), we hypothesized that the generation of at least some circRNAs (e.g., circRNAs characterized by multiple MBNL-binding sites in their flanking introns) would be downregulated by the diminished functional levels of MBNLs, which are sequestered in mutant RNA foci (Miller et al., 2000). To test this hypothesis, we selected 20 well-validated circRNAs and analyzed their expression levels in several experimental systems, including cultured human myoblasts and skeletal muscle biopsy samples from patients and healthy individuals. In addition, we used muscles from the *HSA* ^LR^ transgenic mouse model of DM1 (Mankodi et al., 2000; Sobczak et al., 2013). The analysis of circRNA expression levels was performed with in-house-designed droplet digital PCR (ddPCR) (Hindson et al., 2011; Miotke et al., 2014) assays. We also expanded this analysis and explored global levels of circRNAs using RNA-Seq data from an “exploratory cohort” of DM1 muscle samples of quadriceps femoris (QF) and tibialis anterior (TA) (http://www.dmseq.org/).

In summary, we found no downregulation of the analyzed circRNAs in DM (both DM1 and DM2) samples compared with those in non-DM samples. Therefore, these results question the role of MBNL proteins in circRNA biogenesis in muscles. Interestingly, in our experimental systems that are characterized by a lower level of functional MBNLs, we discovered a consistent increase in circRNA levels. As a result, we identified a subset of circRNAs that were upregulated in DM1 samples and could be used as novel biomarkers. Although the obtained data do not confirm our hypothesis regarding the link between MBNL sequestration and disrupted circRNA biogenesis in DM1 (and DM2), we do not exclude the possibility of the existence of individual circRNAs that are regulated by MBNLs. Additionally, we demonstrated that elevated circRNA levels associate with molecular (alternative splicing) and clinical (muscle weakness) symptoms of DM severity. However, the role of individual circRNAs altered in DM1 and their global function in DM1 pathogenesis remain to be determined.

## Materials and Methods

### Complementary DNA Samples

Four complementary DNA (cDNA) sample sets (**Table 1** and described later) were used in this study. These sets included samples from myoblast cell lines (CL) derived from human skeletal muscles, muscle biopsy (BP) samples from DM1 and DM2 patients and corresponding healthy controls, and samples from the *HSA* ^LR^ transgenic mouse model of DM1 (MM). For the purpose of cDNA generation, total RNA was extracted using the standard protocol, as previously described (Chomczynski and Sacchi, 1987). Reverse transcription was performed according to the manufacturers’ recommendations. All reverse transcription reactions were performed with the use of random hexamers. The particular reverse transcriptases (RTs) used in the analyzed sample sets are indicated later. The DM1-specific splicing aberrations in the muscle sample sets used in this study were evaluated before (Wojciechowska et al., 2018) and are shown (BP_DM2) in **Figure S1**. The splicing aberrations in DM1 samples deposited in the DMseq database and analyzed in this study [see section Analysis of Next-Generation Sequencing Data] were also recently demonstrated (Wang et al., 2018).

**Table 1:** Characteristics of sample sets used in the study.

	Sample set ID	Subset	# of samples
CL	CL_DM1	control	3
		DM1	3
BP	BP_DM1	control	6
		DM1	5
	BP_DM2	control	4
		DM2	9
MM	MM_DM1	control, *FVB*	10
		DM1 model, *HSA* ^LR^	10

CL, human cell lines; BP, human muscle biopsies; MM, mouse muscles.

The sample sets: i) CL_DM1 (generated with SuperScript III RT, Invitrogen, Carlsbad, CA, USA) consisted of three DM1 samples extracted from DM1 myoblast CL (9886, >200 CTG repeats; 10010, >200 CTGs; and 10011, >350 CTGs) and three sex- and age-matched control samples extracted from non-DM myoblast CL (9648, 10104, 10701) as described in Wojciechowska et al. (2014); ii) BP_DM1 (iScript RT, Bio-Rad) consisted of five DM1 and six control QF muscle samples; iii) BP_DM2 (GoScript RT, Promega) consisted of nine DM2 and four control samples, derived from QF or biceps branchii muscles; iv) MM_DM1 (SuperScript III RT, Invitrogen) consisted of 10 DM1-model and 10 control samples of the *HSA* ^LR^ transgenic mouse model of DM1 and control background *FVB* mice, respectively. RNA was extracted from gastrocnemius muscle (Mankodi et al., 2000).

The samples, experimental protocols, and methods reported in this study were carried out in accordance with the approval of the local ethics committees: NRESCommittee.EastMidlands-Nottingham2 and the University of Rochester Research Subjects Review Board. Informed consent was obtained from all subjects.

### Selection of Circular RNAs for Experimental Analyses

Twenty circRNAs (**Table 2**) whose levels were experimentally evaluated in our study were selected from previously detected (Jeck et al., 2013; Memczak et al., 2013; Salzman et al., 2013; Rybak-Wolf et al., 2015; Zhang et al., 2016) circRNAs deposited in circBase (December 2016) (Glazar et al., 2014; http://www.circbase.org/). We considered only circRNAs validated by at least 20 next-generation sequencing (NGS) reads in at least two of the previously mentioned studies. Fourteen circRNAs were selected based on the relatively high level in different types of cells/tissues and a relatively high (≥10% in Jeck et al., 2013) proportion compared with that of their linear counterparts (mRNA). Four circRNAs were selected based on a high number (*n* ≥ 10) of potential MBNL-binding sites (YGCY motifs; Goers et al., 2010) in adjacent (300 nt upstream and 300 nt downstream) sequences of their flanking introns. Two additional circRNAs selected for analysis were circCDR1as and circMBNL1. Additionally, eight circRNAs were experimentally analyzed for the purpose of validation of the most differentiated circRNAs identified based on RNA-Seq data analysis of control and DM1 QF samples (see later). In the mouse sample set, we analyzed seven circRNAs. Five of them, i.e., circCamsap1, circHipk3, circNfatc3, circZkscan1, and circCdr1as, were selected based on orthology to the human circRNAs analyzed in our study (see **Table 2**). Two circRNAs, i.e., circZfp609 and circBnc2, were selected based on their recently reported role in the skeletal muscle (Legnini et al., 2017; Wang et al., 2019).

**Table 2:** CircRNAs selected for analysis.

circRNA	circBase ID	Genome localization (hg 19)	Homing gene	circRNA:mRNA ratio (Jeck et al., 2013)	Number of potential MBNL-binding motifs
**circRNAs selected based on high level in different cells/tissues**					
circASXL1	hsa_circ_0001136	20:30954186|30956926	ASXL1	286%	6
circCASMAP1	hsa_circ_0001900	9:138773478|138774924	CASMAP1	253%	10
circFAM13B	hsa_circ_0001535	5:137320945|137324004	FAM13B	34%	5
circHIPK3	hsa_circ_0000284	11:33307958|33309057	HIPK3	721%	2
circMBOAT2	hsa_circ_0000972	2:9048750|9098771	MBOAT2	19%	5
circMIB1	hsa_circ_0000835	18:19345732|19359646	MIB1	26%	1
circNFATC3	hsa_circ_0000711	16:68155889|68160513	NFATC3	52%	3
circPHC3	hsa_circ_0001359	3:169854206|169867032	PHC3	22%	5
circPIP5K1C	hsa_circ_0000871	19:3660963|3661999	PIP5K1C	11%	2
circSCMH1	hsa_circ_0000061	1:41536266|41541123	SCMH1	23%	3
circSHKBP1	hsa_circ_0000936	19:41089303|41089623	SHKBP1	14%	9
circUBAP2_e7-8	hsa_circ_0001851	9:33971648|33973235	UBAP2	82%	4
circUBAP2_e9-12	hsa_circ_0001847	9:33953282|33963789	UBAP2	63%	3
circZKSCAN1	hsa_circ_0001727	7:99621041|99621930	ZKSCAN1	99%	14
**circRNAs selected based on high number of potential MBNL-binding motifs**					
circCCDC134	hsa_circ_0001238	22:42204878|42206295	CCDC134	98%	10
circFOXK2	hsa_circ_0000816	17:80521229|80526077	FOXK2	26%	12
circPDCD11	hsa_circ_0000258	10:105197771|105198565	PDCD11	129%	11
circPROSC	hsa_circ_0001788	8:37623043|37623873	PROSC	15%	11
**circRNAs additionally included in the analysis**					
circCDR1as	hsa_circ_0001946	X:139865339|139866824	CDR1as	–	5
circMBNL1	hsa_circ_0001348	3:152017193|152018156	MBNL1	–	7

### PCR Assays Design and Validation

For the experimental analysis of selected circRNAs, we designed PCR assays that allowed the amplification and parallel analysis of circRNAs and their linear counterparts. Each assay consisted of one primer common to the circular and linear transcript and two primers specific for either circular or linear transcript. The only exceptions were assays designed for circCDR1as (circRNA generated from a single-exon transcript) and circMBNL1, which consisted of four primers (two for the circular transcript and two for the linear transcript). Primer sequences are shown in **Table S1**.

The PCR products of the designed assays were validated by analysis in agarose gel electrophoresis (the length of each product was as expected). Briefly, PCR was performed in a 10-μl reaction composed of 0.3 μl of a 10-μM dilution of forward and reverse primers (0.6 μl in total; primers were synthesized by Sigma-Aldrich, Saint Louis, MO, USA), 0.125-μl deoxynucleotide triphosphate mix (concentration of each nucleotide was 10 mM) (Promega), 0.05-μl GoTaq DNA Polymerase (concentration 5 u/μl) (Promega), 2-μl 5× colorless GoTaq reaction buffer (containing 7.5 mM MgCl_2_) (Promega), 6.225-μl deionized water, and 1-μl cDNA template. The following cycling conditions were used: 2 min at 95°C, followed by 35 cycles at 95°C for 20 s, 58–60°C (different for individual assays) for 20 s, and 72°C for 20 s, followed by 5 min at 72°C. The obtained PCR products were visualized on a standard 1.5% agarose gel. Additionally, the specificity of each product was confirmed by Sanger sequencing performed on an ABI Prism 3130 genetic analyzer (Applied Biosystems, Carlsbad, CA, USA) according to the manufacturer’s general recommendations.

### Droplet Digital PCR

The level of circRNAs was analyzed with the use of the ddPCR technique (Hindson et al., 2011; Miotke et al., 2014) developed by Bio-Rad. ddPCR involves partitioning the analyzed sample into many low-volume droplet reactions, and only a fraction of these reactions contains one (in most cases) or more template molecules (positive droplets). The final concentration of the analyzed templates was determined by Poisson statistical analysis of the number of positive and negative droplets. ddPCR analyses were performed according to the manufacturer’s general recommendations. Briefly, reactions were carried out in a total volume of 20 μl, containing 10-μl 2× EvaGreen Supermix (Bio-Rad), 1 μl 4 μM forward primer, 1 μl 4 μM reverse primer, and different amounts of cDNA template, determined on the basis of optimization reactions performed for each analyzed gene/transcript. A QX200 ddPCR droplet generator (Bio-Rad) was used to divide the reaction mixture into up to 20,000 droplets. The initial dilution of the cDNA samples ensured that most of the generated droplets contained zero or one template molecule. The thermal parameters of the PCR were as follows: 5 min at 95°C, followed by 40 cycles of 30 s at 95°C, 30 s at annealing temperature (optimized for each gene) and 45 s at 72°C, followed by 2 min at 72°C, 5 min at 4°C, enzyme inactivation at 90°C for 5 min and holding at 12°C. The amplified products were analyzed using a QX200 droplet reader (Bio-Rad). The exact number of cDNA particles (representing particular transcripts) was calculated based on the number of positive (containing template cDNA molecules) and negative (without template cDNA molecules) droplets using QuantaSoft (Bio-Rad) version 1.7.4.019 software, which utilizes Poisson distribution statistics.

In the analyses, we took the factor of the aforementioned cDNA dilution into account. Importantly, in our analysis, we used the following exclusion criteria: i) from the analysis of the level of a particular circRNA, we excluded samples with less than 10 positive droplets corresponding to the linear counterpart of this circRNA; ii) in the individual sample set, we did not consider the analysis of a particular circRNA if more than half of the samples (including DM and control samples) were excluded from the analysis in step i. Additionally, due to the limited amount of RNA samples, not all of the originally selected circRNAs were tested in the BP_DM2 sample set.

For each analyzed circRNA, their levels in particular samples were calculated as a fraction of circular particles (FCP) constituted by the amount of circRNA particles (C) in a total number of particles [circRNAs (C) and their linear counterparts (L)] generated from a particular gene:

(1) FCP=C/(C+L)

The only exception was circCDR1as for which both linear and circular transcripts are generated from the same single exon (PCR primers designed for analysis of linear transcripts are also specific to cDNA generated from circular transcripts). Thus, the equation in this case is as follows:

(2) FCP=C/L

Additionally, the levels of circRNAs and their linear counterparts were normalized against the levels of housekeeping genes (i.e., *ACTB* and *GAPDH*).

### Analysis of Next-Generation Sequencing Data

For the purpose of global analysis of circRNA expression, we used the RNA-Seq data [Gene Expression Omnibus (GSE86356)] deposited in the DMseq database (Wang et al., 2018) (http://www.dmseq.org/). From the data sets of 126 samples derived from different muscle tissues, we chose the data sets of muscles represented by the highest number of samples, i.e., QF and TA. To avoid potential technical variations for analysis, we selected only samples for which sequencing data were generated with uniform procedures. For each sample, paired-end sequencing libraries were prepared from rRNA-depleted total RNA. Reverse transcription was performed using random primers, followed by second strand cDNA synthesis, end repair, adenylation, and ligation of adapters. Sequencing was performed using an Illumina HiSeq 2000 system (Illumina, San Diego, CA, USA), followed by processing with standard HiSeq 2000 software. Reads were mapped to the human genome (GRCh37/hg19) using Hisat2 (Kim et al., 2015). For the analysis, we selected data sets for 23 QF samples (11 control samples and 12 DM1 samples) and 27 TA samples (six control samples and 21 DM1 samples). The GSM accession numbers of selected samples are shown in **Table S2**. The average number of mappable reads in selected samples was ∼29 million (ranging from ∼18 to ∼97 million reads; median ∼26 million reads) and constituted 92% of the total library size on average. The length of reads was 60 nt. The detection and quantification of circRNAs and their linear mRNA counterparts in the selected samples was performed with CIRI2 (Gao et al., 2018), which uses maximum likelihood estimation based on multiple seed matching. This tool enables the identification of back-spliced junction reads and the filtration of false positives derived from repetitive sequences and mapping errors. The normalized level of circRNAs was calculated either as a number of circRNA-specific reads per million mappable reads (RPM) or as a fraction of circRNA-specific reads in a total number of circRNA-specific and corresponding linear reads (FCR). Note that FCR corresponds to FCP calculated based on the number of circular and linear RNA particles. The level of circRNAs was also normalized against the number of reads specific to individual housekeeping genes (e.g., *ACTB* or *GAPDH*).

### Statistical Analyses

All statistical analyses were performed using Statistica (StatSoft, Tulsa, OK, USA) or Prism v. 5.0 (GraphPad, San Diego, CA, USA). All *p*-values were provided for two-sided tests. If necessary, the false discovery ratio (FDR) was calculated according to the Benjamini–Hochberg procedure (http://www.biostathandbook.com/multiplecomparisons.html). To compare the observed and expected (equal occurrence of increases and decreases) proportion of increased and decreased circRNAs in DM samples in particular sample sets or RNA-Seq data sets, we used the chi^2^ test. All human genome positions indicated in this report refer to the February 2009 (GRCh37/hg19) human reference sequence. The functional association analysis of the genes corresponding to circRNAs was performed with the use of DAVID Bioinformatics Resources (Huang da et al., 2009a; Huang da et al., 2009b). The computational prediction of exons in MBNL1 was performed with the GENSCAN online tool (http://genes.mit.edu/GENSCAN.html), using the default filters (i.e., organism: vertebrate; suboptimal exon cutoff: 1.00; print options: predicted peptides only). The exon prediction was performed in the sequence of the second exon of *MBNL1* flanked by directly adjacent 1-kb fragments of downstream and upstream introns (coordinates of analyzed sequence: chr3:152016193-152019155). Correlations of circRNA levels with DM1 severity were performed for TA samples with the use of phenotypic (ankle dorsiflexion force) and splicing alteration data deposited in the DMseq database.

## Results

### Selection of Circular RNA Species for Expression Analysis in DM1

To check whether the level of individual circRNAs is affected in DM1, we selected 20 circRNAs reported in previous studies (Jeck et al., 2013; Memczak et al., 2013; Salzman et al., 2013; Zhang et al., 2014; Rybak-Wolf et al., 2015) and deposited in circBase (Glazar et al., 2014; http://www.circbase.org/). To avoid falsely identified circRNAs, we considered only circRNAs validated by at least 20 NGS reads in at least two previous studies. Fourteen circRNAs (**Table 2**) were selected based on their relatively high levels (compared with other circRNAs) in different types of cells/tissues and relatively high (≥10% in Jeck et al., 2013) expression levels compared with that of their linear counterparts (mRNAs). Four circRNAs (**Table 2**) were selected based on a high number (*n* ≥ 10) of potential MBNL-binding sites (YGCY motifs; Goers et al., 2010) in adjacent (300 nt upstream and 300 nt downstream) sequences of their flanking introns. Additionally, we selected circCDR1as (hsa_circ_0001946) (Hansen et al., 2011; Memczak et al., 2013), the well-studied circRNA generated from the antisense transcript of the *CDR1* gene (CDR1as), and circMBNL1 (hsa_circ_0001348) (**Table 2**), which derives from the second exon of *MBNL1*, which is reportedly involved in the self-regulation of *MBNL1* expression (Konieczny et al., 2017) and linked to circRNA biogenesis (Ashwal-Fluss et al., 2014).

### Design of Assays to Analyze Circular RNA Expression

**Figure 1:**
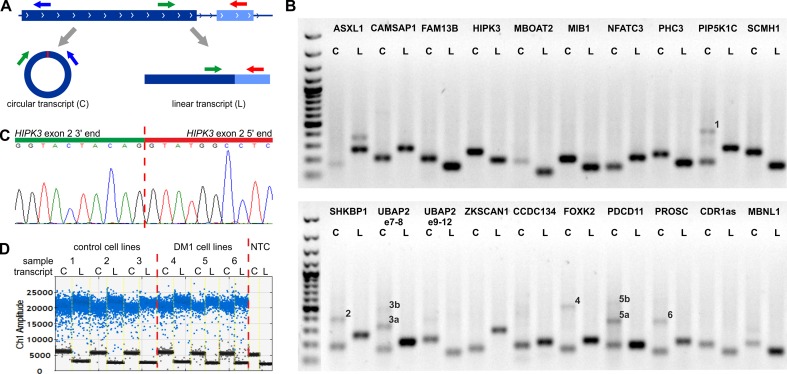
Design and validation of the assays used for analysis of circRNA levels. **(A)** Each assay consisted of three primers as follows: one primer (green arrow) common to the circular (C) and linear (L) transcripts (circRNA and mRNA, respectively) and two primers specific for either circular (blue arrow) or linear (red arrow) transcripts. The primers specific to linear transcripts were located in either the downstream or upstream exon, outside of circRNA-coding exons. **(B)** Gel electrophoresis confirming the size of circRNA-specific (C) and linear, mRNA-specific (L) amplicons. Additional bands in some tracks corresponding to circRNAs indicate the occurrence of circRNA-related concatemers (see You et al., 2015), labeled by numbers on the gel. The expected lengths of the indicated concatemers are: 1–427 bp, 2–383 bp, 3a–314 bp, 3b–473 bp, 4–498 bp, 5a–357 bp, 5b–537 bp, and 6–360 bp. An additional band in *ASXL1* linear transcript track corresponds to the alternative transcript containing alternatively included (97-nt long) exon 5. The first track is the GeneRuler 1-kb DNA Ladder (Thermo Fisher Scientific, Waltham, MA, USA). PCR reaction was performed for a control CL sample from CL_DM1 sample set. **(C)** Exemplary result of Sanger sequencing of the predicted back-splice site of circHIPK3. Results of sequencing of back-splice sites of other circRNAs are shown in **Figure S2**. **(D)** Exemplary result of the ddPCR analysis of circHIPK3 in the myoblast CL (CL_DM1) sample set. Sample number and type are indicated above the graph. NTC—no template control. Ch1 Amplitude—relative fluorescence signal in channel 1. Each blue dot represents one copy of either circular or linear transcript (positive droplets), while the black dots represent negative (empty) droplets. For each sample, the number of positive and negative droplets was used to calculate the concentration of the analyzed transcript.

For each selected circRNA, we designed PCR assays allowing amplification and parallel analysis of a given circRNA and its linear mRNA counterpart. Each assay consisted of three primers as follows: one primer common to both the circular and linear transcripts and two primers specific for either the circular or linear transcript (**Figure 1A**, **Table S1**). The size of circRNA-specific amplicons was analyzed by agarose gel electrophoresis (**Figure 1B**), and the predicted back-splice sites were subsequently confirmed by Sanger sequencing (**Figure 1C** and **Figure S2**). The specific assays were employed for quantification of cDNA copies corresponding to circRNA and linear mRNA transcripts using ddPCR that enables absolute quantification of nucleic acid templates (**Figure 1D**; for details, see Materials and Methods).

Additionally, gel electrophoresis of the PCR product specific for circMBNL1 revealed an additional longer band. Analysis of this additional band led to the identification and characterization of a new circRNA (circMBNL1’) consisting of the second exon of *MBNL1* and a 93-nt fragment of the large (∼114-kb long) downstream intron 2 (**Figure S3**). The analysis of the surrounding sequence with the GENESCAN online tool identified (with high confidence) the incorporated fragment of intron as an exon, with canonical 5’ and 3’ splice sites.

### Analysis of Expression Levels of the Selected Circular RNAs in DM Samples

**Figure 2:**
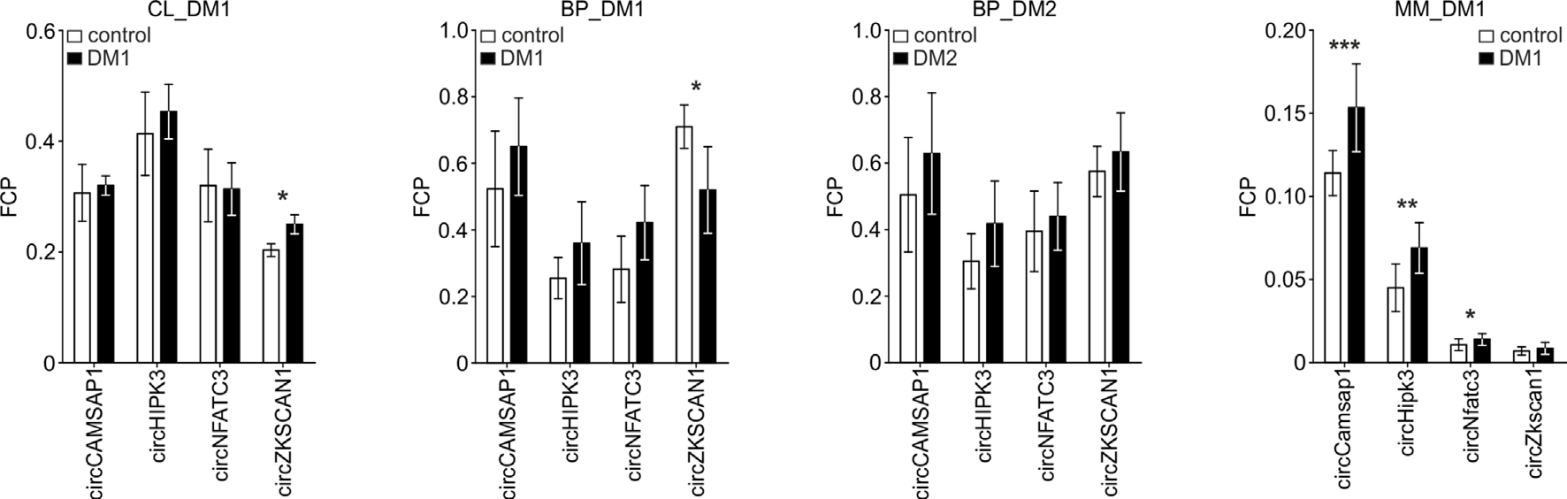
Comparison of circCAMSAP1, circHIPK3, circNFATC3, and circZKSCAN1 levels in DM and control samples. The tested sample sets are indicated above the graphs. The values indicated by white or black bars are the averaged fraction of circular particles (FCPs) calculated for either control or DM samples, respectively. The whiskers represent the standard deviation (SD) values. The differences in circRNA levels were compared with *t*-tests, and the asterisks represent the following significance levels: *p < 0.05; **p < 0.01; ***p < 0.001. The exact *p*-values, SD values, and numbers of samples analyzed are indicated in **Table S3**.

Human myoblast cell lines (CL), as well as skeletal muscle biopsy (BP) tissues from DM1, DM2, and non-DM controls, were used to compare expression levels of circRNAs in DM and unaffected samples in three different sample sets (defined in Materials and Methods and **Table 1**). As shown in **Figure 2**, four circRNAs (i.e., circCAMSAP1, circHIPK3, circNFATC3, and circZKSCAN1) that were consequently analyzed in all sample sets, in all but two cases (circNFATC3 in the CL_DM1 sample set and circZKSCAN1 in the BP_DM1 sample set) showed an increase in DM samples. Also, the other selected circRNAs tend to be rather increased than decreased in DM samples (**Figure S4**) (for details, see **Table 3** and **Table S3**). The marginally significant differences of the individual cicRNA levels are indicated by asterisks on the graphs. A similar effect was observed when the circRNAs levels were normalized against the levels of housekeeping genes (*GAPDH* and *ACTB*; data not shown).

**Table 3:** Results of experimental analyses of circRNA expression levels.

	DM1/DM2 status			
	CL_DM1	BP_DM1	BP_DM2	MM_DM1
**circCAMSAP1**	↑ **(*p* = 0.69)**	↑ **(*p* = 0.27)**	↑ **(*p* = 0.27)**	↑ **(*p* = 0.0009)**
**circHIPK3**	↑ **(*p* = 0.48)**	↑ **(*p* = 0.12)**	↑ **(*p* = 0.14)**	↑ **(*p* = 0.002)**
**circNFATC3**	↓ **(*p* = 0.89)**	↑ **(*p* = 0.06)**	↑ **(*p* = 0.49)**	↑ **(*p* = 0.049)**
**circZKSCAN1**	↑ **(*p* = 0.02)**	↓ **(*p* = 0.03)**	↑ **(*p* = 0.40)**	↑ **(*p* = 0.2)**
circASXL1	↑ (*p* = 0.91)	ex	↓ (*p* = 0.15)	–
circFAM13B	↑ (*p* = 0.31)	↓ (*p* = 0.12)	–	–
circMBOAT2	↓ (*p* = 0.51)	ex	–	–
circMIB1	↓ (*p* = 1.00)	↓ (*p* = 0.91)	–	–
circPHC3	↓ (*p* = 0.55)	↑ (*p* = 0.16)	↑ (*p* = 0.26)	–
circPIP5K1C	↓ (*p* = 0.87)	↑ (*p* = 0.08)	–	–
circSCMH1	↑ (*p* = 0.42)	↑ (*p* = 0.42)	–	–
circSHKBP1	↓ (*p* = 1.00)	ex	–	–
circUBAP2_e9-12	↑ (*p* = 0.09)	ex	–	–
circUBAP2_e7-8	↑ (*p* = 0.66)	↓ (*p* = 0.81)	–	–
circCCDC134	↑ (*p* = 0.55)	ex	–	–
circFOXK2	↑ (*p* = 0.27)	↑ (*p* = 0.08)	–	–
circPDCD11	↑ (*p* = 0.55)	ex	–	–
circPROSC	↑ (*p* = 0.79)	↑ (*p* = 0.27)	–	–
circCDR1as	↓ (*p* = 0.70)	↓ (*p* = 0.15)	–	↓ (*p* = 0.07)
circMBNL1	↑ (*p* = 0.82)	ex	–	–
circBnc2	–	–	–	↑ (*p* = 0.25)
circZfp609	–	–	–	↑ (*p* = 0.0005)
**chi^2^, *p*-value**	**0.17**	**0.4**	**0.1**	**0.06**

↑, circRNA level increased; ↓, circRNA level decreased; ex, excluded from analysis due to low number of positive droplets (see Materials and Methods); –, not analyzed; **bolded**, circRNAs analyzed in all sample sets.

The disadvantage of analysis of human biopsy samples is that they may not always be of homogenous quality (e.g., different sample sources or divergent tissue and/or RNA treatment protocols may result in differences in RNA integrity). Moreover, the limited access to this type of samples and consequently small sample sets does not always allow the detection (with appropriate statistical support) of smaller changes in the levels of analyzed transcripts. Therefore, in the next step, we used cDNA samples from muscles of the commonly used and well-characterized mouse model of DM1 (*HSA* ^LR^, Mankodi et al., 2000) and compared them with samples from control background (*FVB*) mice. For analysis, we selected five mouse circRNAs (circCamsap1, circHipk3, circNfatc3, circZkscan1, and circCdr1as) that are orthologs of the human circRNAs analyzed in this study. Additionally, we analyzed two circRNAs reportedly involved in muscle development, i.e., circZpf609 (ortholog of human circZNF609) and circBnc2 (ortholog of human circBNC2) (Legnini et al., 2017; Wang et al., 2019). As shown in **Table 3** and **Figure S4**, the levels of four out of seven circRNAs tested in mice (i.e., circCamsap1, circHipk3, circNfatc3, and circZfp609) were significantly increased in *HSA* ^LR^ mice.

In conclusion, our experimental analyses show a trend toward some increase of circRNA level in DM (especially supported in the DM1 mouse model).

### Analysis of Circular RNA Levels in DM1 With RNA-Seq Data Sets

CircRNAs selected for the experiments described previously may not be representative, and global circRNA level changes may be too small to be detected with a few circRNAs. Therefore, in the next step, to better evaluate the global circRNA level, we used the RNA-Seq data deposited in the DMseq database (Wang et al., 2018). For the analysis, we selected data sets of muscle samples most frequently represented in the database, QF muscle (11 control samples and 12 DM1 samples) and TA muscle (six control samples and 21 DM1 samples). To avoid potential technical variations in analysis, we selected only samples with sequencing data generated with uniform procedures (for details, see Materials and Methods).

In total, in QF samples, we detected 22,816 distinct circRNAs (“all”; a substantial fraction were confirmed by just a few reads), 4,168 (18%) of which were classified as “validated” (confirmed by at least five reads in at least two samples), and 152 (0.7%) were classified as “common” (present in all or all but one sample of either control or DM1 samples). In the case of TA samples, the “all” group contained 38,403 circRNAs, and the “validated” and “common” groups contained 7,537 (20% of “all”) and 403 (1% of “all”) circRNAs, respectively. As expected, the fraction of known (deposited in circBase and in Maass et al., 2017) circRNAs increased with the level of validation in both QF and TA (**Table 4**, **Table S4**, **Table S5**).

**Table 4:** Number of circRNAs in quadriceps femoris (QF) and tibialis anterior (TA) tissues in different validation groups.

		“All”			“Validated”			“Common”		
		Total	Known	New	Total	Known	New	Total	Known	New
QF	control	11,960	5,085 (42.5%)	6,875 (57.5%)	3,566	2,425 (68.0%)	1,141 (32.0%)	152	135 (88.8%)	17 (11.2%)
	DM1	16,131	6,503 (40.3%)	9,628 (59.7%)	4,078	2,715 (66.6%)	1,363 (33.4%)	152	135 (88.8%)	17 (11.2%)
	control + DM1	22,816	8,319 (36.5%)	14,497 (63.5%)	4,168	2,765 (66.3%)	1,403 (33.7%)	152	135 (88.8%)	17 (11.2%)
TA	control	10,022	4,593 (45.8%)	5,429 (54.2%)	4,515	3,007 (66.6%)	1,508 (33.4%)	403	336 (83.4%)	67 (16.6%)
	DM1	34,720	11,014 (31.7%)	23,706 (68.3%)	7,536	4,614 (61.2%)	2,922 (38.8%)	403	336 (83.4%)	67 (16.6%)
	control + DM1	38,403	11,816 (30.8%)	26,587 (69.2%)	7,537	4,615 (61.2%)	2,922 (38.8%)	403	336 (83.4%)	67 (16.6%)

**Figure 3:**
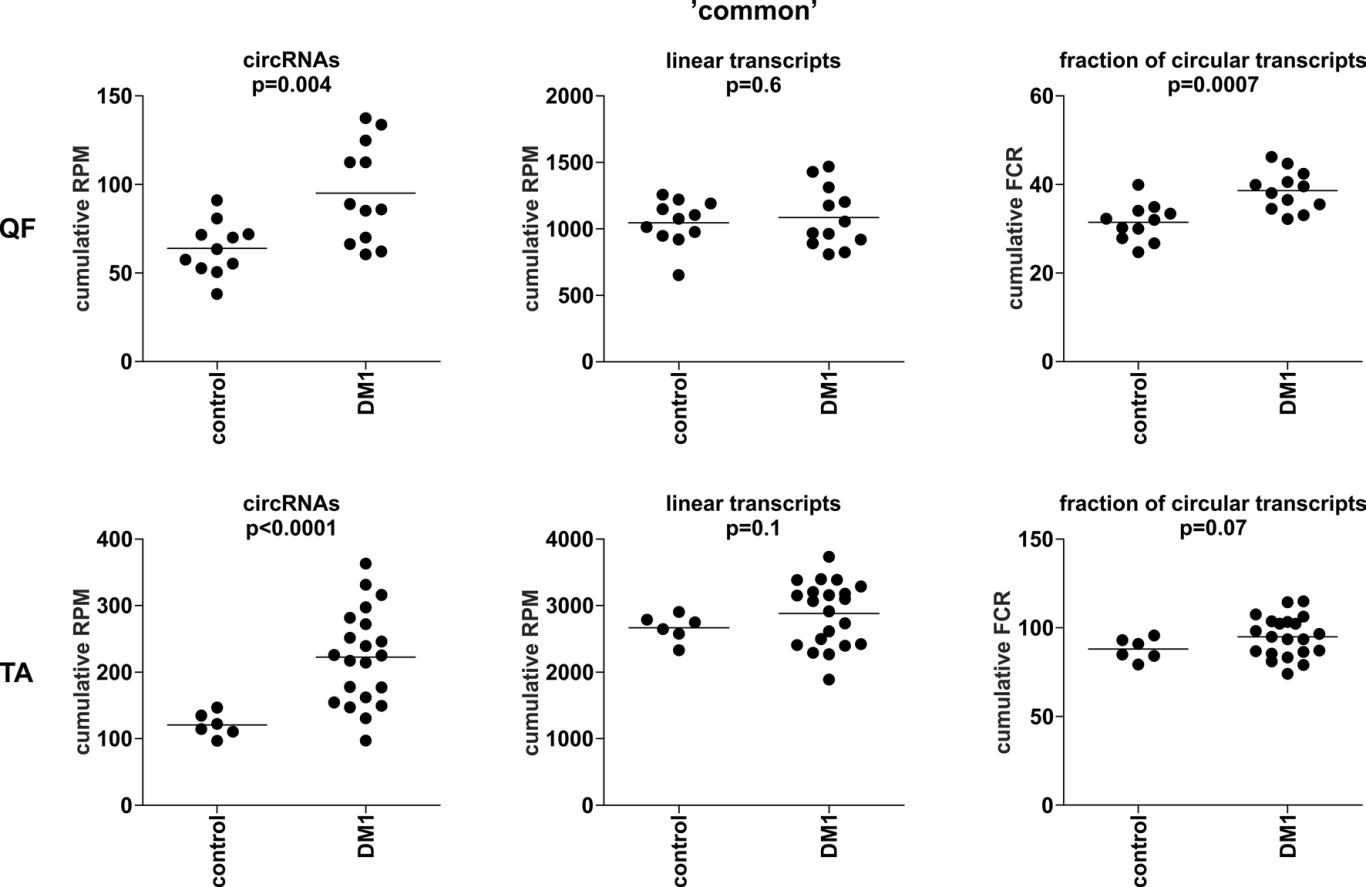
Comparison of cumulative levels of circRNAs and linear RNAs in control and myotonic dystrophy type 1 (DM1) muscle samples. Dot plots depicting the cumulative level of “common” circRNAs and linear transcripts in quadriceps femoris (QF) (upper panel) and tibialis anterior (TA) (lower panel). From the left (in each panel): cumulative reads per million mappable reads (RPM) of circRNAs, cumulative RPM of linear transcripts, and cumulative fraction of circRNA-specific reads in a total number of circRNA-specific and corresponding linear reads (FCR). The false discovery ratio (FDR)-corrected *p*-value (*t*-test with correction for not-equal variance) of the differences between control and DM1 samples is shown above each dot plot.

To compare the global level of circRNA in control and DM1 samples, in each sample, we summarized the number of reads [normalized as reads per million mappable reads (RPMs)] mapping to back-splice sequences (circRNA level) and mapping to the corresponding linear-splice sequences (linear mRNA level). As shown in **Figure 3**, the average global level of “common” circRNAs was significantly increased in DM1 samples (*p* = 0.004 in QF and *p* < 0.0001 in TA). Importantly, no difference was detected compared with corresponding linear transcripts (*p* = 0.6 and *p* = 0.1 in QF and TA, respectively). The increased level of circRNA in DM1 samples was also visible for “all” and “validated” circRNAs (**Figure S5**). Similar results were obtained when the level of transcripts (number of reads) was normalized against the level of individual housekeeping genes, e.g., *ACTB* or *GAPDH* (data not shown).

The previously mentioned changes in circRNA levels may be a reflection of an increase or decrease of expression from a particular gene or genome region. To control for this effect, we also normalized the levels of circRNAs against the levels of their linear counterparts, calculating the level of circRNAs as fraction of circRNA-specific reads in a total number of circRNA-specific and corresponding linear reads (FCR). Again, the cumulative value or averaged FCRs were higher in DM1 samples than in control samples (right graphs in **Figure 3** and **Figure S5**). Additionally, in this analysis, circular transcripts of “common” circRNAs accounted for ∼5–10% of their linear counterparts.

### Differential Expression of Individual Circular RNAs

**Figure 4:**
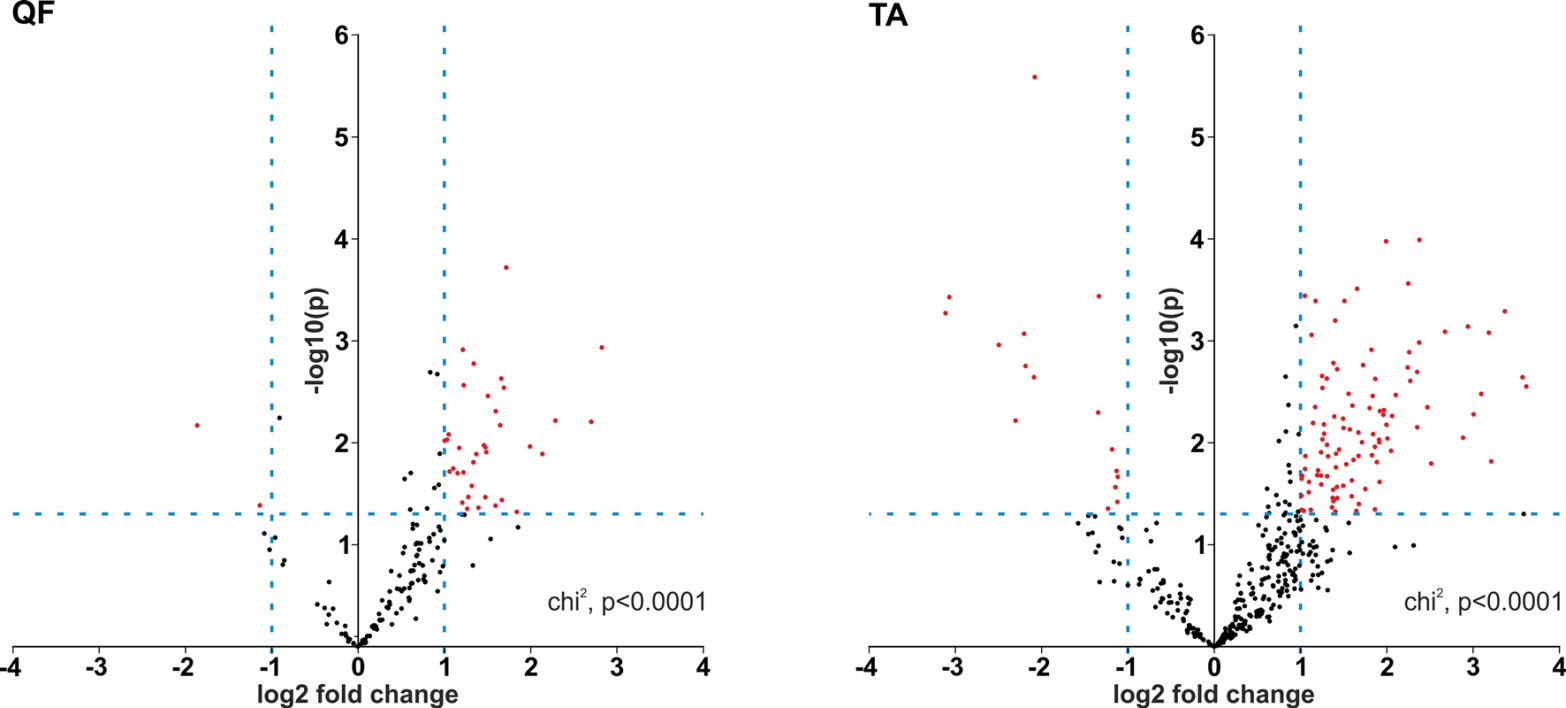
Prevalence of circRNAs with increased levels in DM1. Volcano plots depicting differences in the levels of “common” circRNAs (dots) in DM1 and control samples in QF (left-hand side) and TA (right-hand side). Positive and negative values of log2 fold change indicate increased and decreased circRNAs in DM1. Each red dot represents circRNA fulfilling the following criteria of expression change: *p*-value <0.05 and log2 fold change ≤−1 or ≥1 (the thresholds indicated by dotted lines).

Although it was not the main purpose of the study, by using the generated data, we also analyzed the differential expression of individual circRNAs. This analysis was limited to only the sets of “common” circRNAs (*n* = 152 in QF and *n* = 403 in TA) with expression levels detectable in the vast majority of analyzed samples. The difference in circRNA levels was calculated for the level of circRNAs normalized as RPMs and FCRs of individual circRNAs and expressed as log2 of fold change in DM1 samples vs. control samples. In both QF and TA, the changes in circRNA levels calculated with two normalization methods were highly correlated (**Figure S6**), indicating that circRNA changes do not depend on the expression of genes (level of their primary transcripts) from which they are generated. The results of the analyses are shown in **Table S6** and **Table S7** and graphically summarized in the form of volcano plots (**Figure 4**). The list of circRNAs differentially expressed (RPM value difference at the *p*-value level <0.05) in both QF and TA are shown in **Table 5**; note that four circRNAs are significantly differentiated after adjustment for multiple comparisons in both tissues. Most of the top differentially expressed circRNAs are deposited in circBase, and the majority of them are encoded by exons of known genes (**Tables S4**, **S5**, **S6**, and **S7**). As shown in **Figure 4**, log2 fold change values are substantially shifted toward positive values, indicating an excess of circRNAs with increased levels in DM1 samples. This effect is in line with the global increase in circRNA levels in DM1 (in both QF and TA) described previously. For example, assuming that results fulfilling the following thresholds are significant (*p*-value < 0.05 and log2 fold change ≤ −1 or ≥1), we obtained 38 and 120 differentially expressed circRNAs in QF and TA, respectively. Among these circRNAs, circRNAs with increased expression levels in DM1 (**Figure 4**) were substantially overrepresented [i.e., 36 (95%) in QF (chi^2^, *p* < 0.0001) and 104 (87%) in TA (chi^2^, *p* < 0.0001)]. Similar bias toward circRNAs increased in DM1 may also be seen with other methods of normalization (e.g., such as FCR or normalization against the level of housekeeping genes; data not shown) as well as with other cutoff thresholds. Among circRNAs for which both RPM and FCR values were decreased in DM1, we studied whether MBNL may contribute to their biogenesis. We conducted the analysis of introns (300 nt upstream and 300 nt downstream from circRNA-generating exons) flanking these circRNAs. However, we did not show enrichment of potential MBNL-binding motifs (*n* ranging from 1 to 9, in most cases *n* ≤ 5) that would justify the role of MBNLs in their biogenesis. The interesting exception was circGSE1 (having as many as 30 potential MBNL-binding sites), with decreased RPM and RCF values in DM1 in TA [log2 fold change = −2.1; false discovery rate (FDR)-corrected *p*-value = 0.0001 and log2 fold change = −0.9; FDR-corrected *p*-value = 0.1, respectively].

**Table 5:** CircRNAs differentially expressed in both QF and TA muscles.

circRNA genome localization	Homing gene	circBase ID	QF			TA		
			log2 fold change	*p*-value	FDR-corrected *p*-value	log2 fold change	*p*-value	FDR-corrected *p*-value
**1:200816768|200822623**	**CAMSAP2**	**hsa_circ_0141534**	**0.835**	**0.002**	**0.049**	**0.946**	**0.001**	**0.019**
1:35824526|35827390	ZMYM4	hsa_circ_0011536	1.005	0.010	0.073	1.050	0.0004	0.016
1:59805630|59844509	FGGY	hsa_circ_0006633	0.611	0.020	0.091	1.253	0.009	0.053
10:126628943|126631876	n/a	hsa_circ_0006545	1.264	0.044	0.146	1.426	0.035	0.118
10:34558585|34573173	PARD3	hsa_circ_0018168	1.370	0.013	0.073	1.671	0.008	0.050
**11:33307959|33309057**	**HIPK3**	**hsa_circ_0000284**	**0.917**	**0.002**	**0.049**	**0.831**	**0.008**	**0.049**
2:110919180|110920712	NPHP1	hsa_circ_0056019	1.718	0.0002	0.029	1.052	0.018	0.078
2:215632206|215646233	BARD1	hsa_circ_0001098	1.596	0.005	0.064	2.942	0.001	0.019
2:240929491|240946787	NDUFA10	hsa_circ_0001118	2.287	0.006	0.064	1.240	0.026	0.096
21:30693542|30702014	BACH1	hsa_circ_0001181	1.277	0.034	0.133	1.602	0.004	0.039
3:170906491|170912424	TNIK	hsa_circ_0002387	1.225	0.003	0.049	1.319	0.014	0.064
3:196118684|196129890	UBXN7	hsa_circ_0001380	1.031	0.009	0.073	1.175	0.0004	0.016
6:158703295|158735300	n/a	hsa_circ_0142312	1.103	0.018	0.091	1.673	0.013	0.064
**6:158733083|158735300**	**n/a**	**hsa_circ_0142313**	**1.215**	**0.001**	**0.049**	**1.921**	**0.005**	**0.040**
**6:170846322|170858201**	**PSMB1**	**hsa_circ_0078784**	**1.661**	**0.002**	**0.049**	**1.960**	**0.005**	**0.041**
7:18705836|18706099	HDAC9	hsa_circ_0007904	1.667	0.036	0.138	1.013	0.022	0.089
7:80418622|80440017	SEMA3C	hsa_circ_0004365	1.994	0.011	0.073	2.048	0.012	0.061
7:99621042|99621930	ZKSCAN1	hsa_circ_0001727	0.606	0.045	0.146	0.636	0.049	0.143
8:52773405|52773806	PCMTD1	hsa_circ_0001801	1.051	0.008	0.073	1.129	0.001	0.019
9:37424842|37426651	GRHPR	hsa_circ_0001861	1.647	0.007	0.064	1.373	0.035	0.118

**bolded**, circRNAs differentially expressed in QF and TA at the level of FDR-corrected p-value < 0.05.

The functional association analysis of genes corresponding to circRNAs either increased or decreased in DM1 in TA (67 distinct genes at *p* < 0.01 for differences in RPM, **Table S7**) showed the strongest association (enrichment) with the following UniProt keywords: “phosphoprotein” [number of involved genes (*n*) = 46, fold enrichment (FE) = 1.7, Benjamini corrected *p*-value (*p*_BC_) = 0.0005] and “alternative splicing” (*n* = 52, FE = 1.5, *p*_BC_ = 0.001; **Figure S7**). The genes were also associated with the Gene Ontology cellular component (CC) term “nucleoplasm” (*n* = 24, FE = 2.5, *p*_BC_ = 0.004; **Figure S7**). A similar analysis performed for QF (18 distinct genes) also showed an enrichment of genes associated with alternative splicing and nucleus localization keywords/terms among the top results (**Figure S7**), but the associations were nonsignificant due to the much smaller number of analyzed genes.

### Identification of Multi-circRNA Genes

**Figure 5:**
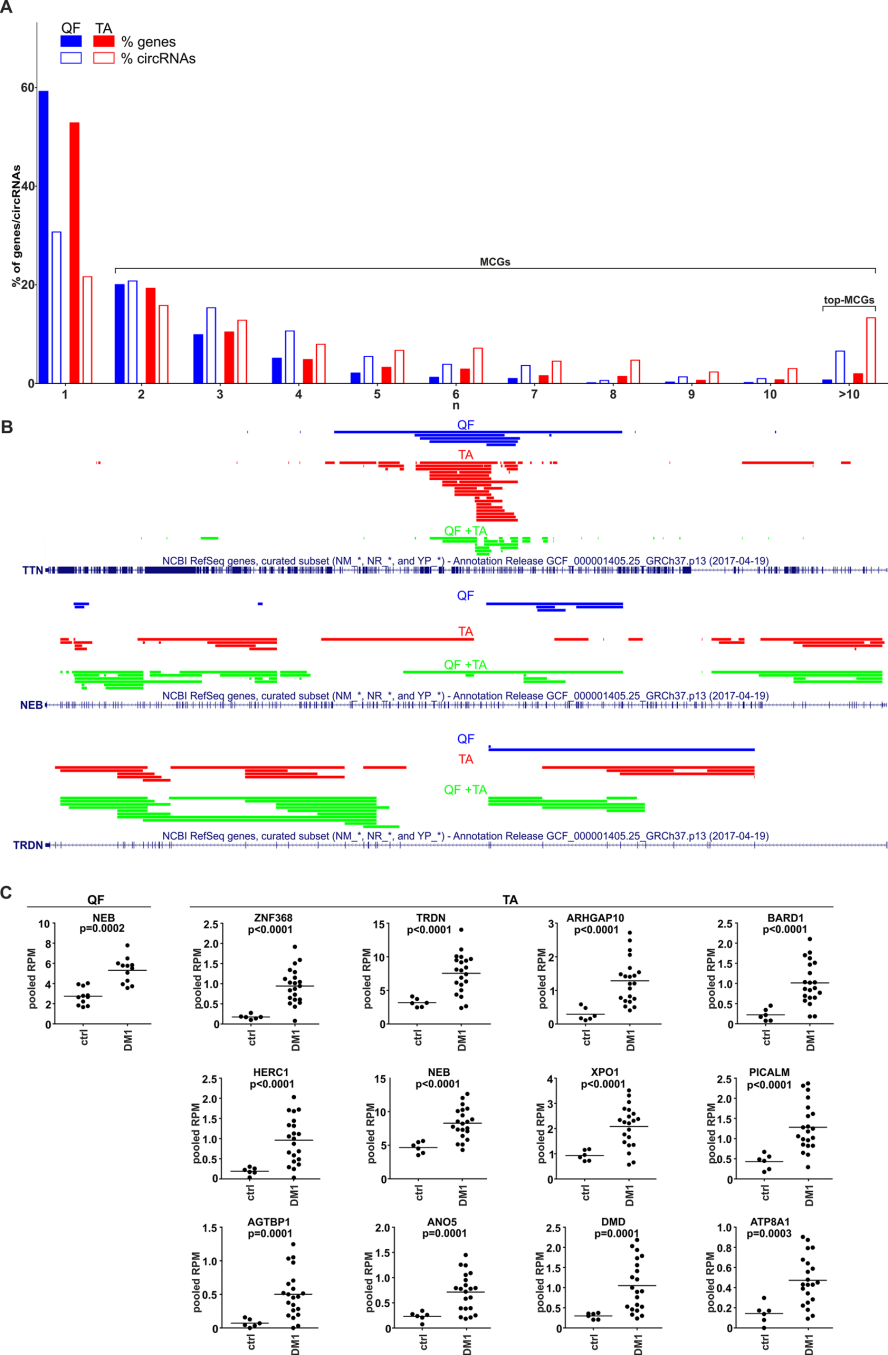
CircRNAs generated from multi-circRNA genes (MCGs). **(A)** Bar graph showing the percentage of genes that generate a particular number (*n*) of distinct circRNA species (solid bars) and the percentage of circRNAs generated from these genes (empty bars). Blue and red bars represent QF and TA, respectively. For example, in QF, the genes generating more than 10 circRNAs constitute ∼1% of all circRNA-generating genes but generate ∼7% of all circRNAs. **(B)** The maps of *TTN*, *NEB*, and *TRDN* (RefSeq tracks) with schematic representation of regions (color lines) overlapping exons giving rise to circRNAs (presented with the use of University of California—Santa Cruz Genome Browser). Blue, red, and green lines represent circRNAs specific to QF, specific to TA, common to QF and TA, respectively. **(C)** Dot plots depicting levels (pooled RPMs) of top-MCG-specific circRNA pools most profoundly differentiated between control (ctrl) and DM1 samples in QF and TA. The FDR-corrected *p*-value is shown above each graph. In each graph, each dot represents pooled circRNA-specific RPM values in the individual sample.

During the analysis, we noticed that a substantial number of circRNAs were generated from multi-circRNA genes (MCGs), which give rise to more than one circRNA. Furthermore, 14 MCGs in QF and 59 MCGs in TA (top-MCGs) generated more than 10 distinct circRNAs. As shown in **Figure 5A** (empty bars), cumulatively 69 and 78% of circRNAs were generated from MCGs, and 7 and 13% were generated from top-MCGs in QF and TA, respectively. The top-MCGs from which the highest numbers of circRNAs were generated were *titin* (*TTN*: 44 circRNAs in QF and 86 circRNAs in TA; cumulatively 96 distinct circRNA species), *nebulin* (*NEB*: 41 and 59; cumulatively 66), and *triadin* (*TRDN*: 24 and 37; cumulatively 39). All three genes are strongly related to biological functions and highly expressed in skeletal muscles. Other top-MCGs strongly related to the function of skeletal muscles are* dystrophin* (*DMD*), *myopalladin*, *myomesin 1*, and *myosin IXA*. Notably, the previously mentioned muscle-related multiexon MCGs were strongly enriched in new (not present in circBase) circRNAs (∼95 vs 34%/39% in all “validated” circRNAs in QF/TA samples). This finding may have been observed because skeletal muscle tissues were not comprehensively studied (reported in the circBase) in the context of circRNA discovery.

The maps of genomic regions giving rise to circRNAs generated from top-MCGs common to QF and TA are shown in **Figure 5B** and **Figure S8**. As shown in the figures, the back-splice sites of almost all circRNAs overlapped with the splice sites of canonical exons; therefore, almost all circRNAs may derive from the sequences of canonical exons. Moreover, a substantial fraction of circRNAs were common to QF and TA (green lines, QF + TA), and tissue-specific circRNAs mostly resulted from the higher number of circRNAs detected in TA. Interestingly, in most cases, circRNA-annotated sequences were not randomly distributed and clustered in the center of the gene. The effect was especially visible for circRNAs common to QF and TA. The most profound example of this distribution was *TTN*. The opposite example was *NEB* in which circRNA-annotated sequences were more or less randomly distributed over the entire gene. The observed distributions do not indicate that circRNAs are preferentially generated from exons flanked by long introns (Jeck et al., 2013).

### The Level of Circular RNA Pools Generated From Particular Multi-circRNA Genes Increases in DM1

Considering circRNAs as competing regulators of linear transcripts, any circRNA generated from a particular gene may affect its linear-transcript-dependent expression. Therefore, in the next step, we compared the cumulative level of circRNAs generated from particular top-MCGs (circRNA pools) in control and DM1 samples. As shown in **Table S8** and **Table S9**, the cumulative RPM value of circRNA pools increased in DM1 samples in 11 out of 14 and 59 out of 59 top-MCGs in QF and TA, respectively. Similar results were also obtained for pooled FCRs (**Table S8** and **Table S9**), as well as for circRNA pools obtained with the other methods of circRNA level normalization (e.g., against the level of housekeeping genes; data not shown). In eight cases (i.e., *GBE1*, *SMARCC1*, *BIRC6*, *SENP6*, *CHD2*, *MYBPC1*, *MAP4K3*, and *RALGAPA2*), the circRNA pools were increased, although none of the individual circRNAs constituting these pools were significantly differentiated. The levels of the most profoundly differentiated circRNA pools (FDR-corrected *p*-value <0.0005) in QF and TA are shown in **Figure 5C**.

### Circular RNA Levels Are Associated With DM Severity

**Figure 6:**
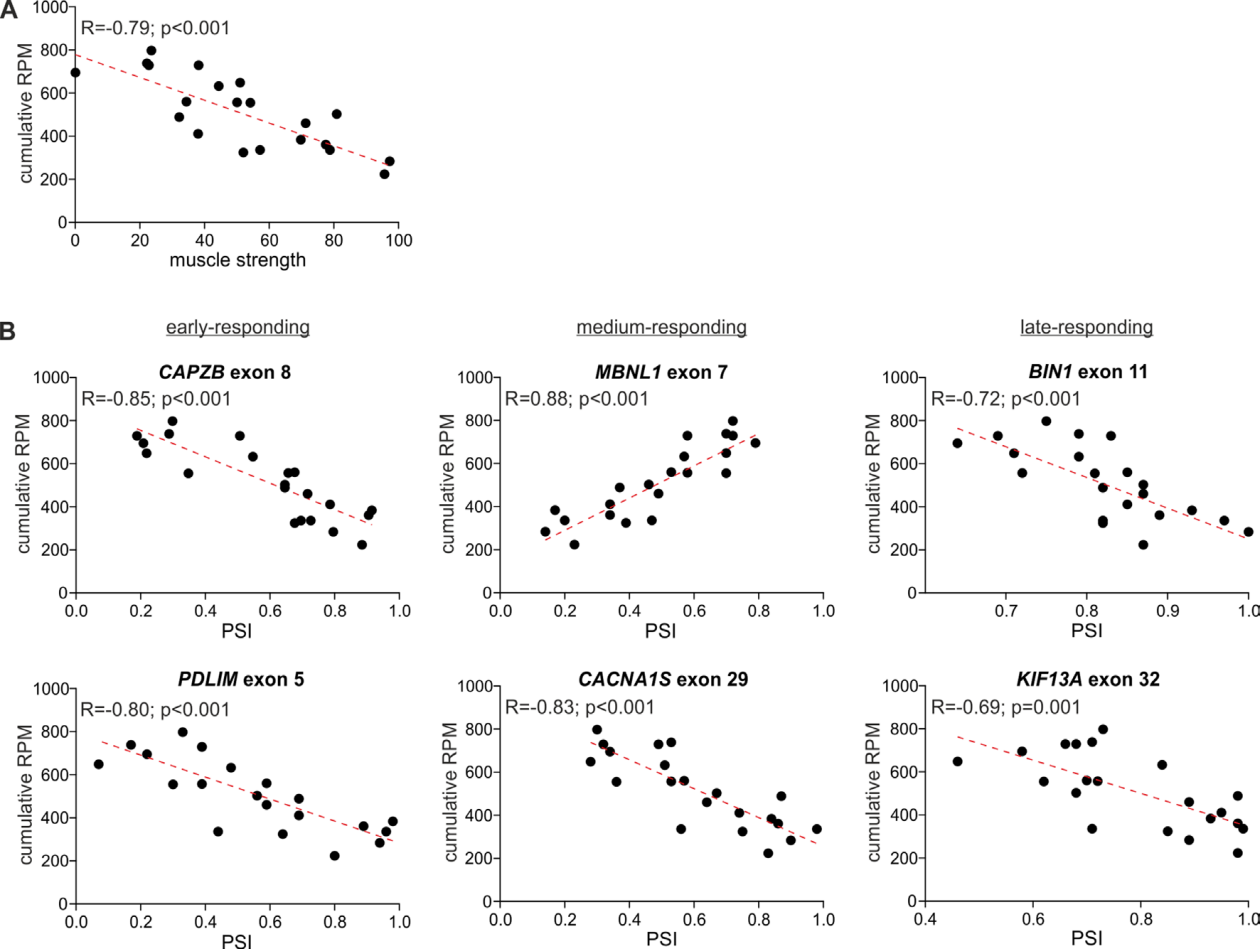
Correlation of global circRNA level with disease severity. For each plot, the *R*-value, *p*-value, and the trendline (red-dotted line) are shown. **(A)** A scatter plot showing the correlation of global circRNA levels normalized as RPMs (*Y*-axis) and muscle strength (*X*-axis). **(B)** Scatter plots showing correlations of global circRNA levels (*Y*-axis) and PSI values of early-, medium-, and late-responding exons alternatively spliced in DM1 (X-axis). Each dot represents an individual TA sample.

The comparison of the global circRNA level in TA with a phenotypic biomarker of muscle strength (ankle dorsiflexion force) associated with DM1 severity showed a substantial correlation [correlation coefficient (*R*) = −0.85; *p* < 0.001]. Significant negative correlation with muscle strength (*p* < 0.05; *R* < −0.434) showed also 117 (out of 403) individual “common” circRNAs and 42 (out of 59) top-MCGs-specific circRNA pools (**Figure 6**, **Table S10**).

In the next step, we compared the circRNA level with the level of early-, medium-, and late-responding alternatively spliced exons, being molecular biomarkers of DM1 severity. As shown in **Figure 6** and **Table S10**, the global circRNA level was significantly correlated with the percent spliced-in (PSI) values of all analyzed exons. The strongest correlation showed exon 7 of *MBNL1* (*R* = 0.88; *p* < 0.001), exon 8 of *CAPZB* (*R* = −0.85; *p* < 0.001), exon 29 of *CACNA1S* (*R* = −0.83; *p* < 0.001), and exon 22 of ATP2A1 (*R* = −0.82; *p* < 0.001). Negative correlations were obtained for exons alternatively excluded in DM1. In contrast, exon 7 of *MBNL1* and exon 7 of *NFIX*, both alternatively included in DM1, showed positive correlations. Similar correlations were obtained for a substantial fraction of individual circRNAs, as well as for the top-MCG-specific circRNA pools (**Table S10**).

## Discussion

Splicing aberrations induced by functional inactivation of MBNL-splicing factors constitute a main pathomechanism of DM1. Previous research suggested that in addition to a function in alternative splicing, MBNL proteins participate in the biogenesis of circRNA, bringing circRNA-flanking introns closer together and facilitating back-splicing (circularization) (Ashwal-Fluss et al., 2014). Thus, downregulation of circRNAs would be expected in DM1 (and in DM2) cells in which expanded CUG (CCUG in DM2) repeats attract MBNLs, leading to their sequestration.

To test whether circRNA levels are changed in DM1 and to verify the role of MBNLs in the biogenesis of circRNA, we analyzed the expression level of up to 20 circRNAs in myoblast CL and skeletal muscle samples derived from patients with DM1 and healthy controls. Among the selected circRNAs were those with a relatively high number (*n* ≥ 10) of potential MBNL-binding motifs in flanking introns, as well as circMBNL1, which is regulated by MBNL1 (Konieczny et al., 2017). Additionally, circCDR1as and circHIPK3, the highly expressed and most extensively studied circRNAs, were among the selected circRNAs (Hansen et al., 2011; Memczak et al., 2013; Zheng et al., 2016). None of the circRNAs tested in our analysis showed a consistent decrease of level in DM1. There was also no decrease in the levels of circRNAs in muscles from patients with DM2 or in muscles from the transgenic mouse model of DM1. All of the previous results question the role of MBNLs as important factors in circRNA biogenesis in muscles. The discrepancy between our study and earlier reports may be because previous analyses were performed in artificial models (artificially generated circRNA genes) in which some of the tested processes (e.g., interaction of MBNLs/Mbl with artificial, usually shorter introns) may take place differently, and the stoichiometry of interacting proteins and RNA particles may be different from those in natural mammalian tissues. Additionally, the previous experiments were mostly performed with the fly Mbl splicing factor. Potentially, human orthologs may not have the exact same circRNA-generation activity, and we cannot exclude the possibility that decreased levels of MBNLs, although they induce aberrations in alternative splicing, are still sufficient for circRNA processing. Furthermore, it is possible that MBNLs play a role in the biogenesis of specific individual circRNAs, which were not tested experimentally in our study. CircGSE1, flanked by multiple MBNL-binding motifs and decreased in DM1, may be an example of such a circRNA. MBNL1-dependent biogenesis of circGSE1 may be additionally supported by the fact that, contrasted with other circRNAs, its increased level is associated with lower DM1 severity (**Table S10**). Another example of circRNA decreased in DM1 and associated with lower DM1 severity is circFGFR1 (**Table S10**). In contrast to our original hypothesis, the previously mentioned experiments showed a trend toward a global increase in circRNA levels in DM1 samples. Although changes in levels of individual circRNAs are small and nonsignificant in most cases, circRNAs with increased levels in DM samples were prevalent in most of our experiments. Additionally, analysis of a higher number of samples from mouse skeletal muscles that provided a better statistical power to detect smaller changes in circRNA levels showed that four out of the seven tested circRNAs were significantly increased in the mouse model of DM1, including circHipk3 regulating cell growth and differentiation (Zheng et al., 2016) and protein-coding circZfp609 playing role in myogenesis (Legnini et al., 2017; Wang et al., 2019).

To check whether the global circRNA level is indeed increased in DM1, we used publicly available RNA-Seq data sets deposited in the DMseq database (http://www.dmseq.org/). The advantage of such data is that they are generated by an independent experimenter blind to the hypotheses tested in particular studies (also in ours). The increased global level of circRNA in DM1 was confirmed in two independent sets of samples, consisting of samples from two different skeletal muscles, QF and TA.

CircRNAs, generated either cotranscriptionally or posttranscriptionally (Wilusz and Sharp, 2013; Ashwal-Fluss et al., 2014; Kramer et al., 2015), compete with their linear counterparts (mRNAs) for their shared linear precursor (pre-mRNA). However, notably, some circRNAs are the main or exclusive products generated from their precursors (e.g., circCDR1as). The generation of circRNA may be a mechanism of mRNA downregulation (Ashwal-Fluss et al., 2014). Alternatively, disturbances and delays in mRNA maturation may increase the duration of the immature transcript and shift the balance of pre-mRNA processing in favor of circRNA biogenesis (Ashwal-Fluss et al., 2014; Zhang et al., 2016; Liang et al., 2017). In DM1, such disturbances in transcript maturation may be caused by the sequestration of MBNLs and aberrations in splicing. The increased global level of circRNA in DM1 may simply be a side effect of splicing aberrations or secondary effect of the chronic pathological state of DM1, not dependent on MBNL1 or splicing alterations.

Furthermore, as the levels of circRNAs are altered in such disorders as Duchenne muscular dystrophy (DMD) or dilated cardiomyopathy (Khan et al., 2016; Legnini et al., 2017), it may suggest that deregulation of circRNAs is generally associated with a muscle pathological state. It may be supported by the results of recent studies demonstrating changes of circRNA levels in different muscle diseases and physiological conditions. For example, it was shown that several circRNAs [e.g., circZNF609 (Legnini et al., 2017; Wang et al., 2019), circQKI (Legnini et al., 2017), circBNC2 (Legnini et al., 2017), circFGFR4 (Li et al., 2018a), and circFUT10 (Li et al., 2018b)] are differentiated in different muscle conditions and may be involved in the regulation of myoblast proliferation and muscle cell development (well reviewed in Greco et al., 2018).

Also, there are some facts that may link elevated global levels of circRNA or increased levels of an individual circRNA with DM1 pathogenesis. First, the global circRNA level, as well as the levels of substantial fractions of MCG-specific circRNA pools and individual circRNAs were negatively correlated with molecular and clinical biomarkers of DM1 severity. Second, gene ontology analysis of the circRNA genes that were increased in DM1 showed enrichment of the aberrant splicing, phosphoprotein, and nucleoplasm terms. It seems particularly interesting considering that aberrant alternative splicing is one of the most prominent molecular markers of DM1 (Philips et al., 1998; Wang et al., 2007) and it is linked with hyperphosphorylation of CUGBP1 protein (Kuyumcu-Martinez et al., 2007; Wang et al., 2009). Additionally, as recently shown (Ketley et al., 2014; Wojciechowska et al., 2014), utilization of kinase inhibitors alleviated some of the molecular symptoms of DM1, among others, diminishing the nuclear fraction of mutant DMPK transcripts (Ketley A et al., IDMC-11, San Fransico 2017). Third, DM pathogenesis may be also linked with the increase of circZfp609, which was observed in our study. It was recently shown that the level of circZfp609, as well as the level of its human ortholog (circZNF609), is increased in proliferating myoblasts and is downregulated during myogenesis (decreased in more differentiated muscle cells). Functional tests demonstrated that circZfp609/circZNF609 plays a role in promoting myoblast proliferation (possibly by sponging miR-194-5p) (Legnini et al., 2017; Wang et al., 2019). This suggests that an increased level of circZfp609/circZNF609 may delay muscle differentiation and maturation. An increase of circZNF609 was also observed in DMD cells (Legnini et al., 2017) that suggests a link between both dystrophies, i.e., DM and DMD. Finally, recent results by Voellenkle et al. showed that the levels of four-out-of-nine-tested circRNAs were significantly increased in DM1 patients and correlated with muscle weakness (Voellenkle et al., 2019).

By using generated circRNA data sets, we also performed analyses of individual circRNAs and MCG-specific circRNA pools. The analyses led to the identification of many circRNAs and circRNA pools that were significantly differentiated between DM1 and control samples. In both types of analyses and in both analyzed tissues, there was a substantial excess of circRNAs or circRNA pools in DM1. This finding is consistent with the observation of the global increase in circRNA levels in DM1 samples. Although many of the changes in circRNA and circRNA pools reached statistical significance (*p* < 0.05, even after FDR correction), whether the differentiated circRNAs/circRNA pools are specific and biologically relevant to DM1 or result from a global increase in circRNA levels in DM1 cannot be established. One hint as to the role of circRNAs in DM1 may be found in the functional association analysis, which showed that terms related to alternative splicing and nuclear localization were among the strongest associations of genes giving rise to differentiated circRNAs. Other links between aberrations in circRNA levels and DM1 pathogenesis come from the observed associations between circRNA levels and muscle weakness, as well as between circRNA levels and abnormalities of alternative splicing of well-known DM biomarkers. Additionally, the transcripts of at least 10 (*DMD*, *KIF1B*, *MYBPC1*, *NEB*, *NCOR2*, *PICALM*, *RERE*, *SMARCC1*, *UBAP2*, and *USP25*) out of 63 identified top-MCGs were previously shown to be aberrantly spliced in DM1 (Du et al., 2010; Nakamori et al., 2013). Nonetheless, the changes in individual circRNAs require further experimental validation. Moreover, notably, the power of this analysis is limited due to the depth of coverage (adjusted for mRNA analysis) that does not allow reliable estimation of low-level circRNAs.

Interestingly, among the top-MCGs, there are genes highly expressed and strongly associated with the biological function of skeletal muscles [e.g., *TTN* (total number of circRNAs generated in both QF and TA, *n* = 96), *NEB* (*n* = 66), *TRDN* (*n* = 39), *DMD* (*n* = 33), *myopalladin* (*n* = 22), *myomesin 1* (*n* = 18), *or myosin IXA* (*n* = 14)]. All of these genes are large multiexon genes, including *DMD* (2.1 Mbp, up to 81 exons), the largest human gene, and *TTN* (0.3 Mbp, up to 362 exons), which has the highest number of exons (**Figure 5B** and **Figure S8**). A large number of exons increase the number of potential splicing donor/acceptor pairs, which may facilitate the generation of different circRNAs. Alternatively, the higher number of circRNAs generated from multiexon genes may also result from higher chances/numbers of aberrations occurring during processing of their transcripts.

In conclusion, our results indicate that MBNL deficiency does not cause the expected decrease in circRNA levels in DM1 cells and tissues. In contrast, the global level of circRNAs is elevated in DM1. However, the role of the increased level of circRNAs in the pathogenesis of DM1 is unknown and requires further investigation.

## Contribution to the Field Statement

Recently, a great deal of interest has been focused on a new class of RNA molecules called circular RNAs (circRNAs). To date, thousands of circRNAs have been found in different human cells/tissues. Although the function of circRNAs remains mostly unknown, circRNAs have emerged as an important component of the RNA–RNA and RNA–protein interactome. Thus, intensive efforts are being made to fully understand the biology and function of circRNAs, especially their role in human diseases. As an important role in the biogenesis of circRNA may be played by MBNL-splicing factors, in this study, we used DM1 (to a lesser extent, DM2) as a natural model in which the level of MBNLs is decreased. In contrast to the expected effect, our results consistently showed a global increase in circRNA levels in DM1. As a consequence, whole genome transcriptome analysis revealed dozens of circRNAs with significantly altered (mostly increased) levels in DM1. Furthermore, we observed that the circRNA levels were in many cases strongly associated with DM1 severity.

## Data Availability

Publicly available datasets were analyzed in this study. This data can be found here: http://www.dmseq.org/.

## Ethics Statement

The samples, experimental protocols, and methods reported in this study were carried out in accordance with the approval of the local ethics committees: NRESCommittee.EastMidlands-Nottingham2 and the University of Rochester Research Subjects Review Board. Informed consent was obtained from all subjects.

## Author Contributions

KC—designed assays for circRNA identifications and testing, performed all experimental analyses, interpreted the results, performed statistical analyses, prepared figures, tables, and supplementary materials, and wrote the manuscript; KT—participated in data interpretation, performed alternative splicing analysis, participated in manuscript preparation, and prepared cDNA samples; APi—participated in data interpretation, performed alternative splicing analysis, and participated in manuscript preparation; KS—provided CL and patient tissue samples and participated in data interpretation and the manuscript preparation; KK—performed computational circRNA identification and analysis; APh—supervised computational analyses and participated in the manuscript preparation; SS—provided patient tissue samples and participated in the manuscript preparation; JDB—provided patient and mouse tissue samples and participated in the manuscript preparation; MW—provided CL and patient tissue samples and participated in data interpretation and the manuscript preparation; PK—conceived and coordinated the study, supervised the experiments and statistical analyses, interpreted the results, and wrote the manuscript. All authors read and approved the final manuscript.

## Funding

This work was supported by the National Science Center PL [2016/21/N/NZ5/00508 (to KC), 2014/15/B/NZ2/02453 (to KS), 2014/15/D/NZ2/02305 (to AP), 2017/24/C/NZ1/00112 (to KT), and 2014/13/B/NZ5/03214 (to MW)]; Muscular Dystrophy UK [17GRO-PG12-0149 (to JDB)]; Wellcome Trust [Seeding Drug Discovery grant number 107562/Z/15/Z; 2015 (to JDB)]; and Myotonic Dystrophy Support Group (to JDB).

## Conflict of Interest Statement

The authors declare that the research was conducted in the absence of any commercial or financial relationships that could be construed as a potential conflict of interest.
