# Supplementary material for: Global Increase in Circular RNA Levels in Myotonic Dystrophy
Source: Front Genet. 2019 Jul 18;10:649. doi: 10.3389/fgene.2019.00649 (PMC6689976; doi:10.3389/fgene.2019.00649)
Supplement: Supplementary file 11 [file DataSheet_1.pdf]

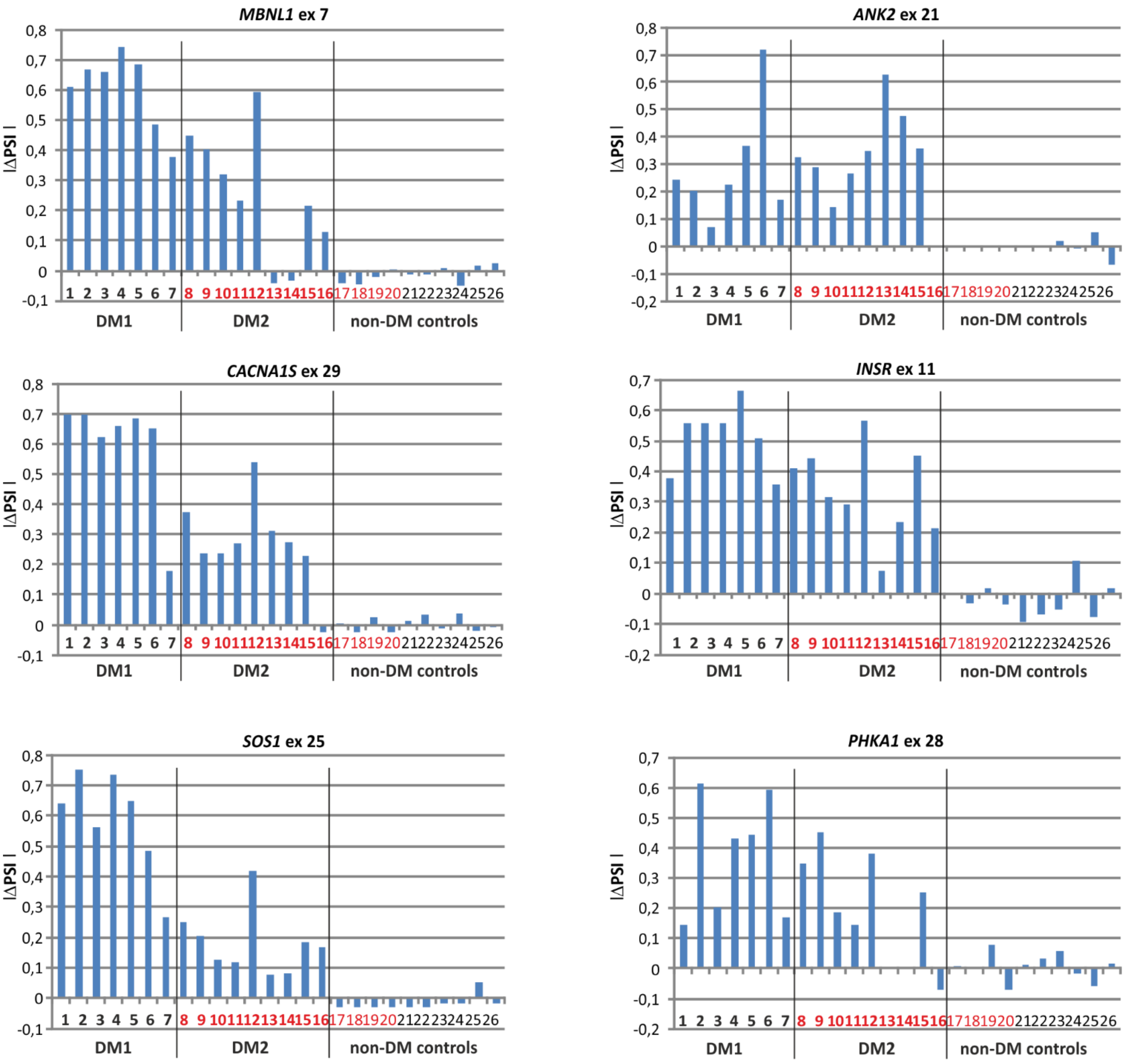

Figure S1. Splicing changes of alternative exons of six genes in DM1 (samples 1-7) and DM2 (samples 8-16) compared to non-DM muscle samples. For each DM sample the deltaPSI was calculated based on MLPA-based splicing assays described before (Wojciechowska et al., 2014). Samples included in BP_DM2 sample sets are indicated in red.

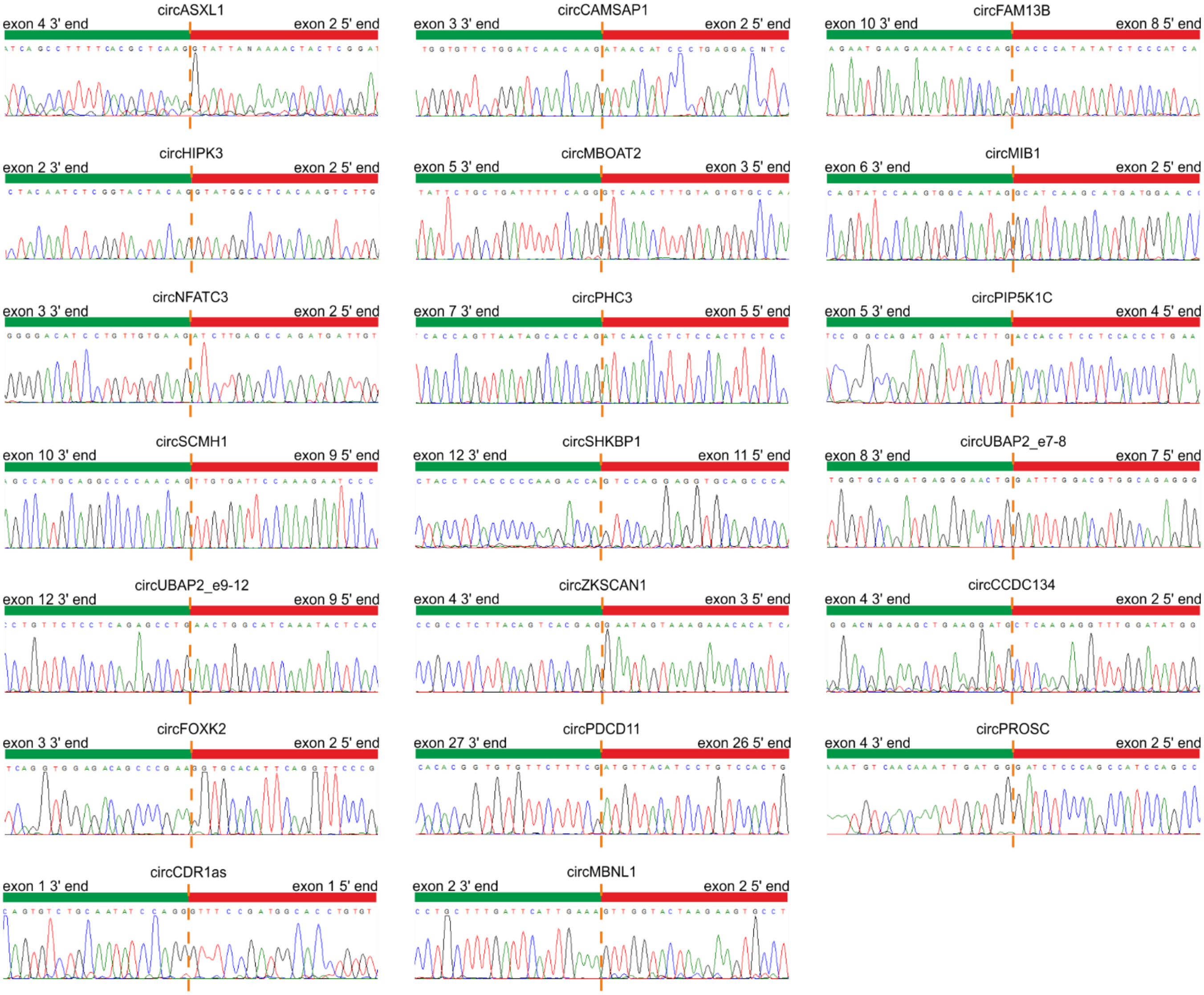

Figure S2. Results of Sanger sequencing of the predicted back-splice sites of the selected circRNAs.

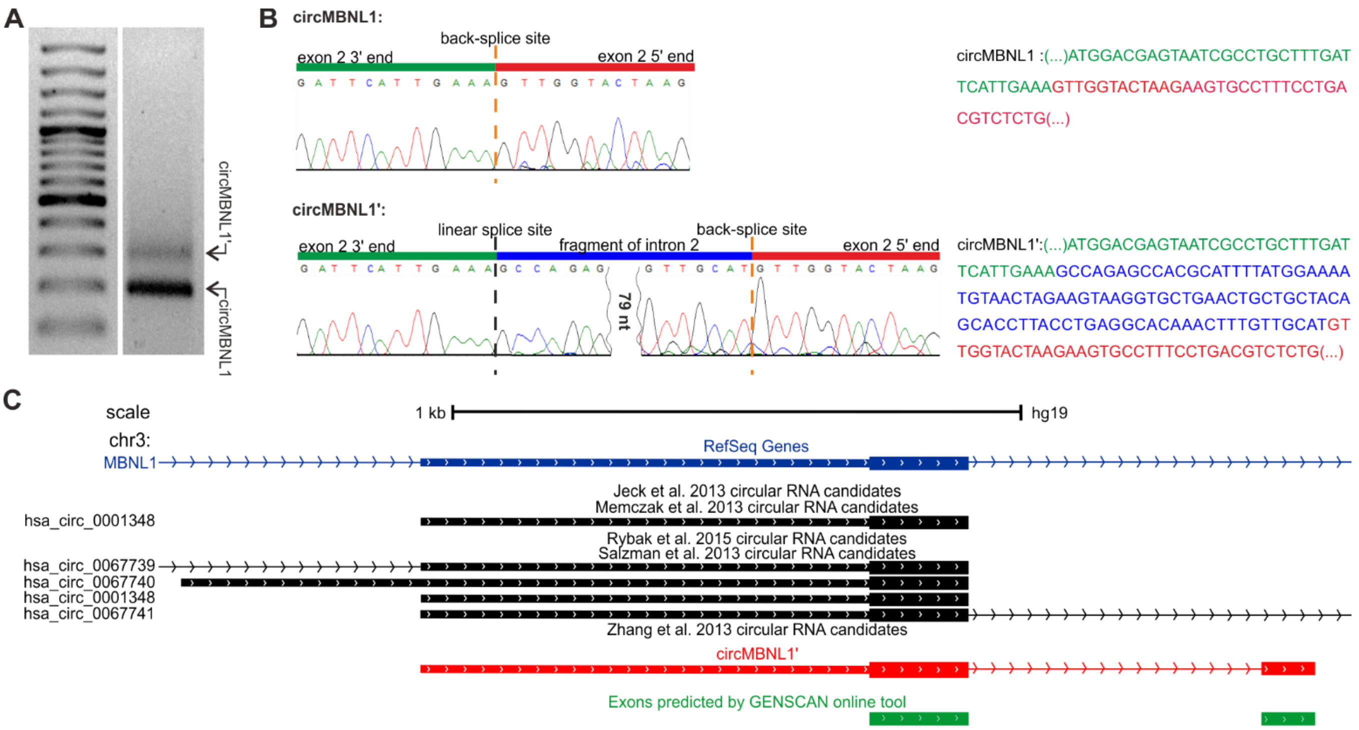

Figure S3. Identification of a new circRNA derived from the *MBNL1* gene. **A)** Agarose gel electrophoresis of the product of PCR performed with the use of circMBNL1-specific primers (right-hand side track). Lower and upper bands represent known circMBNL1 (hsa_circ_0001348) and the newly identified circMBNL1', respectively. The size of the upper band does not correspond to a potential concatemer. The left-hand side track is the GeneRuler 1 kb DNA Ladder (Thermo Fisher Scientific). **B)** The results of the Sanger sequencing of PCR products corresponding to upper and lower bands. Sequencing of the lower band confirmed the expected circMBNL1 back-splice site. However, sequencing of the upper band revealed an additional 93-nt long fragment of the downstream intron. The sequences of both circRNAs are shown to the right. **C)** The map showing the localization of MBNL1 circRNAs. The blue (RefSeq) track shows a fragment of *MBNL1* overlapping exon 2. Black tracks indicate known circRNAs deposited in the circBase. The red track indicates the localization of newly identified circMBNL1'. The green track depicts exons predicted with high confidence by the GENSCAN online tool.

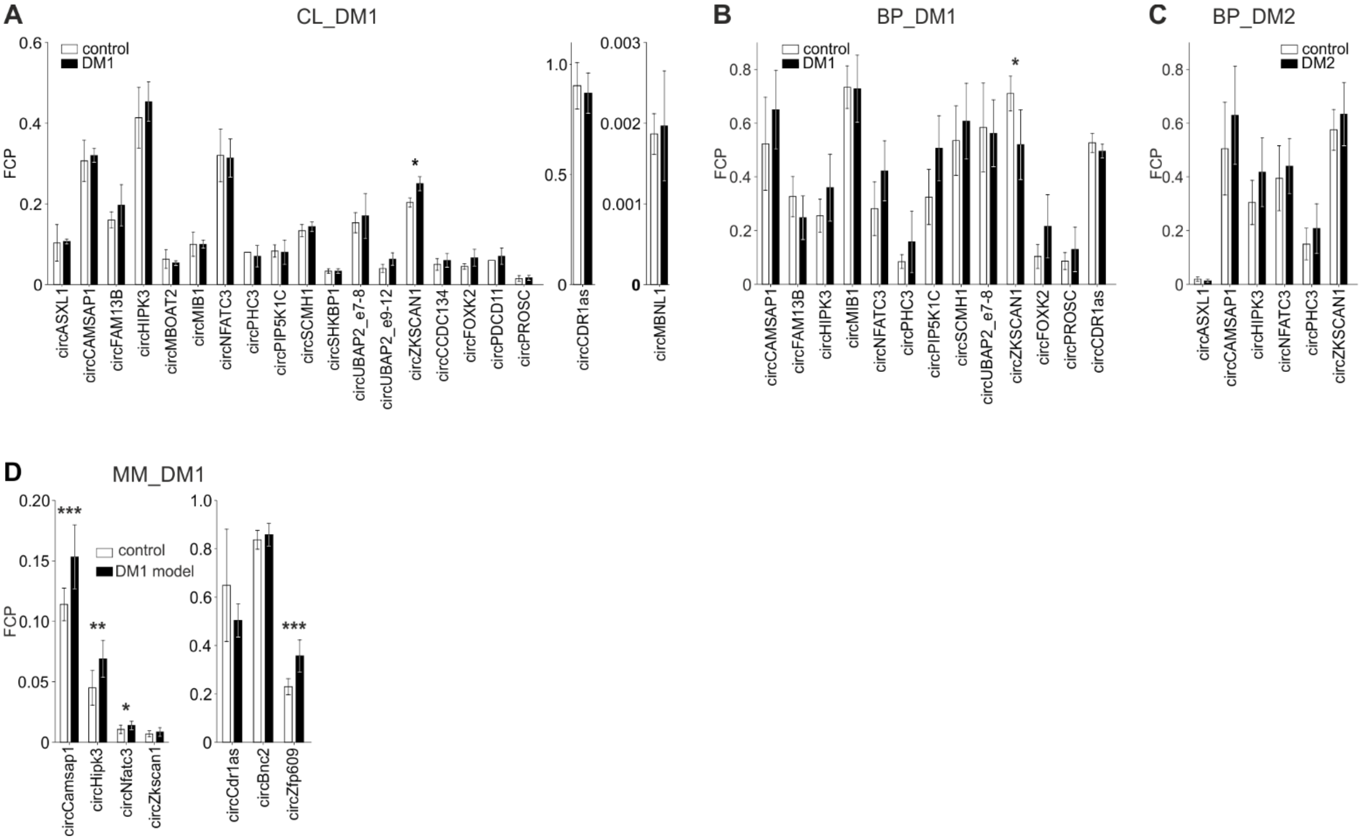

Figure S4. Bar graphs showing the results of circRNA expression analysis performed with the use of different sample sets. **A)** CL_DM1, **B)** BP_DM1, **C)** BP_DM2, **D)** MM_DM1. The scheme of the bar graphs is similar to that used in Figure 2.

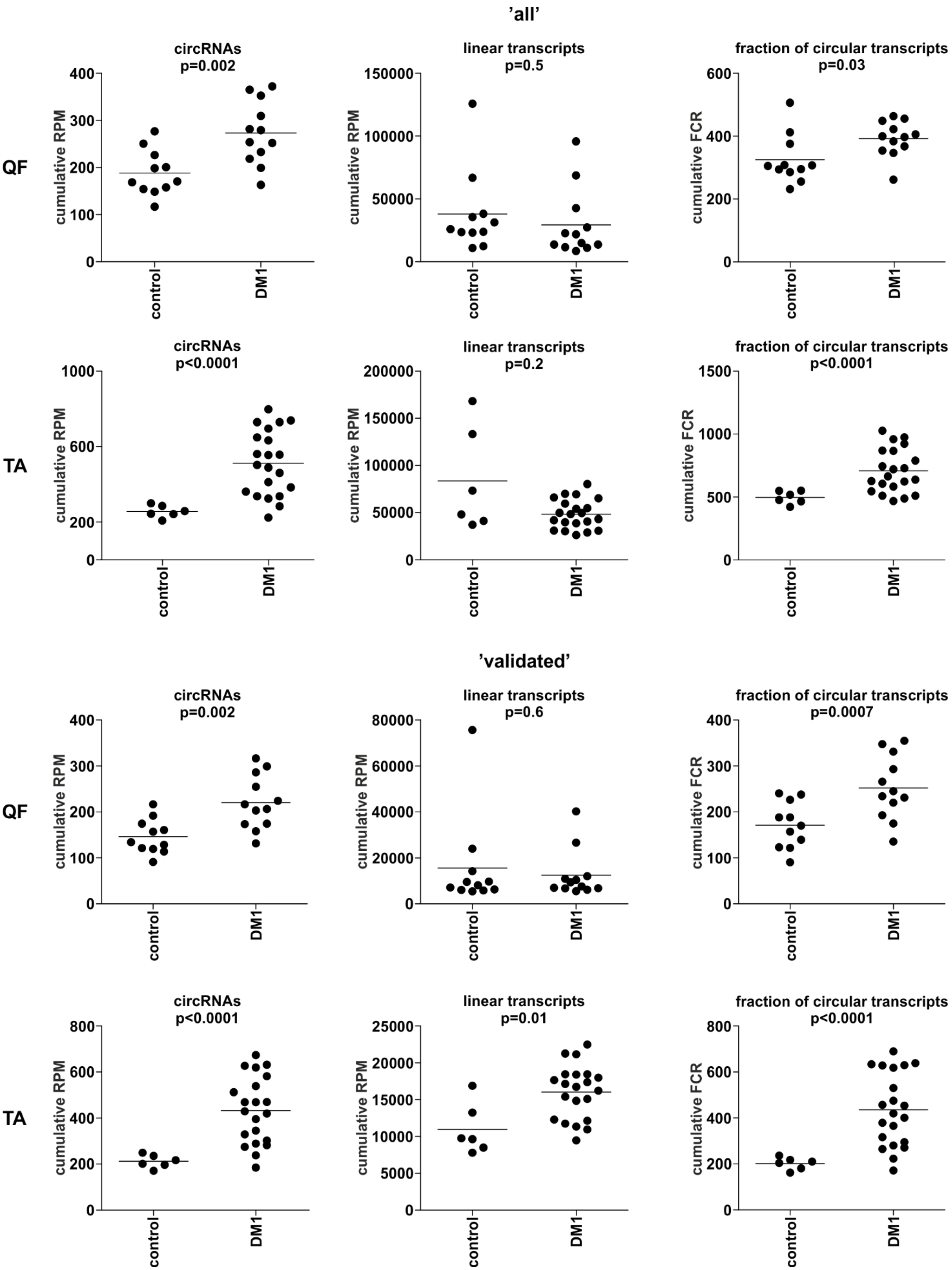

Figure S5. Dot plots depicting the cumulative level of 'all' (upper panels) and 'validated' (lower panels) circRNAs in control and DM1 samples of QF and TA. The scheme of the panels is the same as that used in Figure 3.

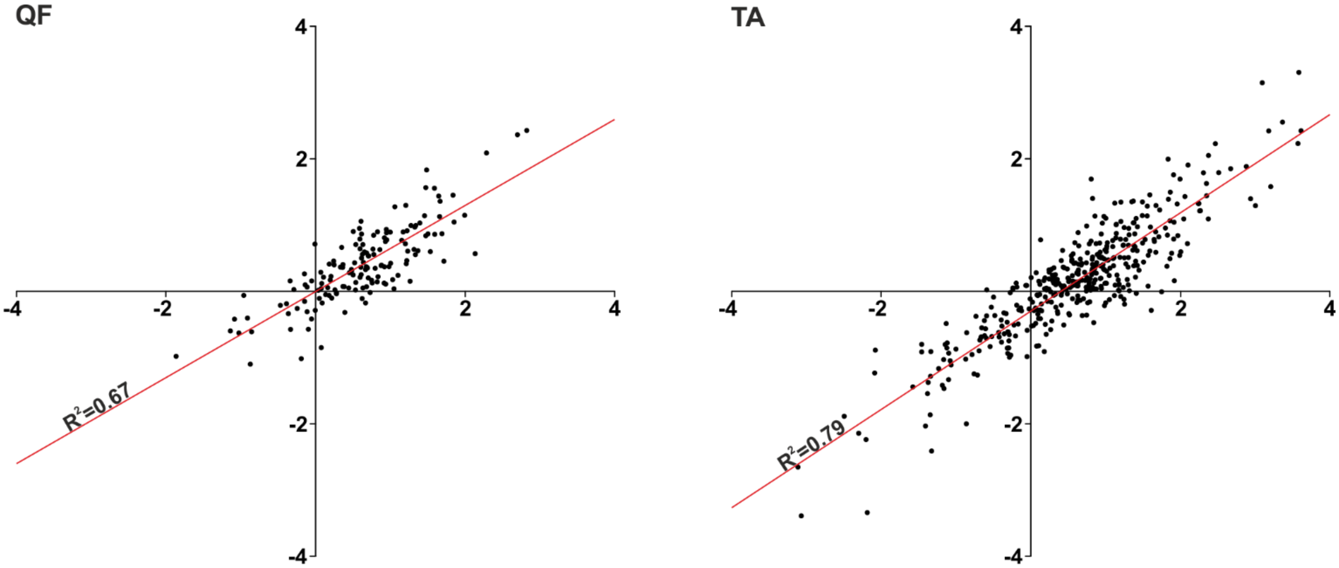

Figure S6. Scatter plots showing correlations of circRNA levels normalized as RPMs (axis X) and FCRs (axis Y). For both QF (on the left-hand side) and TA (right-hand side) samples, the R^2 values are shown above the trendline (red line). Each dot represents an individual circRNA.

UniProt

QF

Sublist	Category	Term	RT	Genes	Count	%	P-Value	Fold Enrichment	Benjamini
☐	UP_KEYWORDS	Phosphoprotein	RT		15	83,3	4,6E-4	2,1	4,0E-2
☐	UP_KEYWORDS	Isopeptide bond	RT		5	27,8	1,2E-2	5,1	4,2E-1
☐	UP_KEYWORDS	Alternative splicing	RT		14	77,8	3,2E-2	1,5	6,2E-1

TA

Sublist	Category	Term	RT	Genes	Count	%	P-Value	Fold Enrichment	Benjamini
☐	UP_KEYWORDS	Phosphoprotein	RT		46	68,7	3,5E-6	1,7	5,4E-4
☐	UP_KEYWORDS	Alternative splicing	RT		52	77,6	1,4E-5	1,5	1,1E-3
☐	UP_KEYWORDS	Isopeptide bond	RT		13	19,4	2,3E-4	3,5	1,2E-2
☐	UP_KEYWORDS	Nucleus	RT		31	46,3	3,4E-4	1,8	1,3E-2
☐	UP_KEYWORDS	Acetylation	RT		23	34,3	6,9E-4	2,1	2,1E-2

cellular component

QF

Sublist	Category	Term	RT	Genes	Count	%	P-Value	Fold Enrichment	Benjamini
☐	GOTERM_CC_DIRECT	cytoplasm	RT		10	55,6	3,0E-2	1,9	7,6E-1
☐	GOTERM_CC_DIRECT	nucleoplasm	RT		7	38,9	3,4E-2	2,5	5,6E-1

TA

Sublist	Category	Term	RT	Genes	Count	%	P-Value	Fold Enrichment	Benjamini
☐	GOTERM_CC_DIRECT	nucleoplasm	RT		24	35,8	2,9E-5	2,5	3,5E-3
☐	GOTERM_CC_DIRECT	sarcoplasmic reticulum membrane	RT		4	6,0	2,2E-4	33,5	1,3E-2

Figure S7. Summary of the functional association analysis showing the most significant enrichment results for UniProt and Gene Ontology “cellular component” categories in the list of genes differentiated in QF and TA.

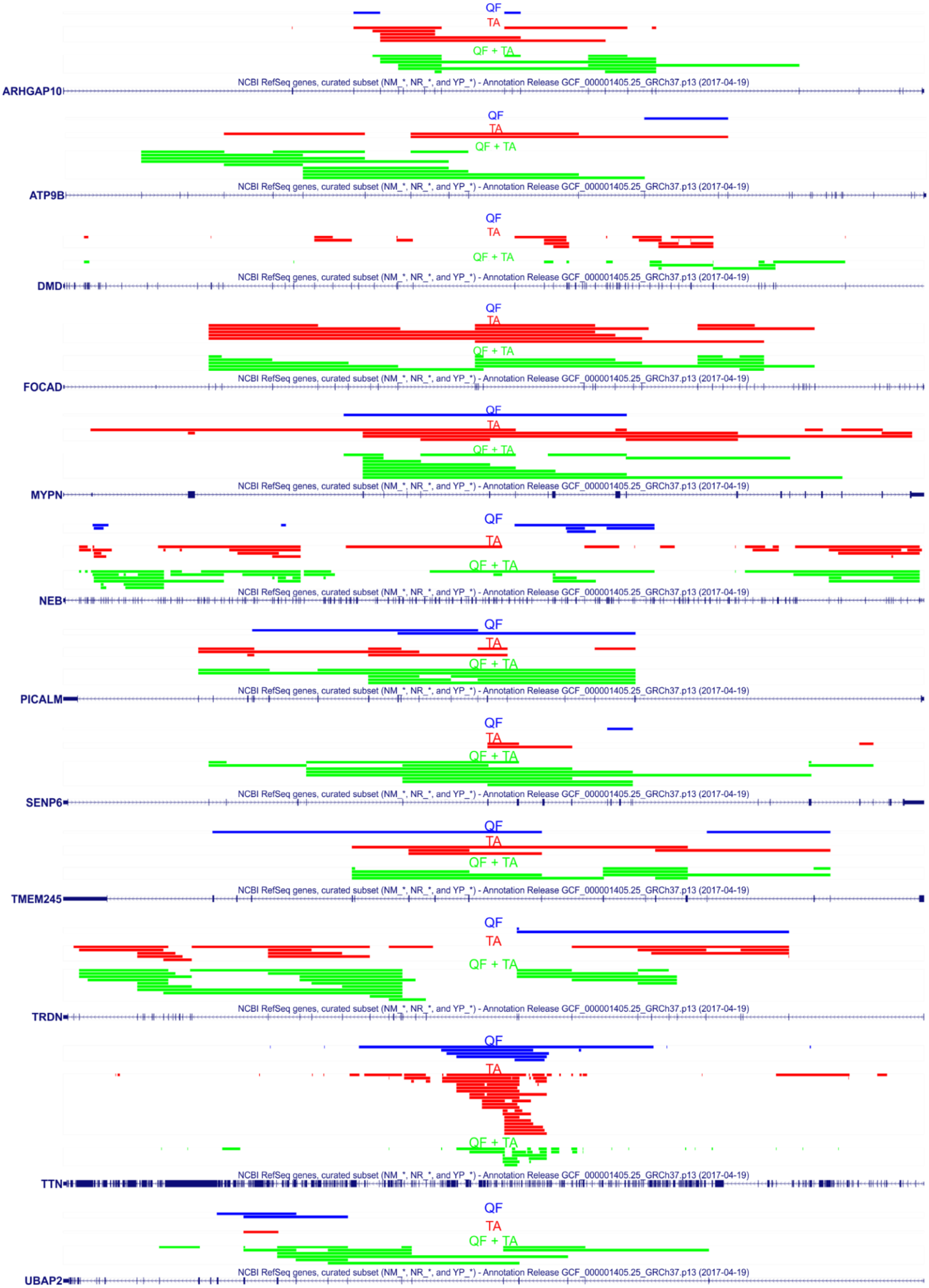

Figure S8. The maps of genomic regions of the top-MCGs in which both QF and TA consequently generate more than ten distinct circRNA species. The colors of the tracks are shown in Figure 5B.
